# Synthesis of Large‐Area MXenes with High Yields through Power‐Focused Delamination Utilizing Vortex Kinetic Energy

**DOI:** 10.1002/advs.202202748

**Published:** 2022-08-17

**Authors:** Qingxiao Zhang, Runze Fan, Weihua Cheng, Peiyi Ji, Jie Sheng, Qingliang Liao, Huirong Lai, Xueli Fu, Chenhao Zhang, Hui Li

**Affiliations:** ^1^ Shanghai Key Laboratory of Rare Earth Functional Materials and Education Ministry Key Laboratory of Resource Chemistry Shanghai Normal University Shanghai 200234 P. R. China

**Keywords:** 2D Ti_3_C_2_T*_x_* MXene, fluid–solid coupling simulation, large nanosheets, liquid‐phase delamination, power‐focused delamination

## Abstract

Evaluating the delamination process in the synthesis of MXenes (2D transition metal carbides and nitrides) is critical for their development and applications. However, the preparation of large defect‐free MXene flakes with high yields is challenging. Here, a power‐focused delamination (PFD) strategy is demonstrated that can enhance both the delamination efficiency and yield of large Ti_3_C_2_T*_x_* MXene nanosheets through repetitive precipitation and vortex shaking processes. Following this protocol, a colloidal concentration of 20.4 mg mL^–1^ of the Ti_3_C_2_T*_x_* MXene can be achieved after five PFD cycles, and the yield of the basal‐plane‐defect‐free Ti_3_C_2_T*_x_* nanosheets reaches 61.2%, which is 6.4‐fold higher than that obtained using the sonication–exfoliation method. Both nanometer‐thin devices and self‐supporting films exhibit excellent electrical conductivities (≈25 000 and 8260 S cm^‐1^ for a 1.8 nm thick monolayer and 11 µm thick film, respectively). Hydrodynamic simulations reveal that the PFD method can efficiently concentrate the shear stress on the surface of the unexfoliated material, leading to the exfoliation of the nanosheets. The PFD‐synthesized large MXene nanosheets exhibit superior electrical conductivities and electromagnetic shielding (shielding effectiveness per unit volume: 35 419 dB cm^2^ g^–1^). Therefore, the PFD strategy provides an efficient route for the preparation of high‐performance single‐layer MXene nanosheets with large areas and high yields.

## Graphical abstract

A new method for the preparation of large 2D Ti_3_C_2_T*_x_* MXene nanosheets is reported. The method is based on conventional etching followed by repetitive precipitation and vortex shaking process, which efficiently transfer the mechanical energy for exfoliation. Consequently, large defect‐free sheets that show an excellent electromagnetic shielding performance are produced with high yields.

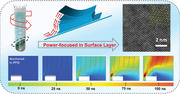

## 1 Introduction

The family of MXenes (general formula: M*_n_*_+1_X*_n_* T*_x_*, M is transitional metal, X is carbon or nitrogen, T*_x_* represents surface terminations, *n* = 1–3) is one of the latest additions to 2D materials composed of transition‐metal nitrides, carbides, or carbonitrides.^[^ ^1^ ^]^ These novel 2D materials have a broad range of promising applications in various fields, including energy storage,^[^ ^2^ ^]^ catalysis,^[^ ^3^ ^]^ superconductivity,^[^ ^4^ ^]^ and electromagnetic shielding.^[^ ^5^ ^]^ To date, about 30 distinct MXenes materials have been reportedly prepared in the laboratory.^[^ ^1b^ ^]^ The past decade has witnessed a growing interest in the exploration of these fascinating ultrathin 2D materials owing to their unique physical and chemical properties, including metallic conductivity,^[^ ^6^ ^]^ presence of abundant surface groups, and the ability to form solid solutions (both with respect to the metal and C or N). These advantageous properties further widen the application scope of these ultrathin 2D materials.^[^ ^1b^ ^]^ One of the earliest and most widely studied MXenes is Ti_3_C_2_T*_x_*, which is generally obtained from the MAX (general formula: M*_n_*_+1_AX*_n_*, where M is a transition metal, A is mostly a IIIA and IVA element, and X is C and/or N, and *n* = 1–3) phase precursor Ti_3_AlC_2_ in two steps: selective etching and intercalation.^[^ ^1a^ ^]^ The unit cell of Ti_3_AlC_2_ contains a Ti_6_C octahedron (M_6_X) stacked between layers of Al atoms. The chemical stability of the amphoteric Al layer is relatively weak compared to that of the strong Ti_3_C_2_ layer. Therefore, the Al atomic layer can be selectively removed from the MAX phases by using etching reagents such as acids,^[^ ^1^, ^2^, ^7^ ^]^ bases,^[^ ^8^ ^]^ or molten salts.^[^ ^9^ ^]^ After the removal of the Al atomic layers via etching with HF, the 2D nanomaterial Ti_3_C_2_T*_x_* (where T*_x_* represents the surface terminations =O, —OH, and —F) with an inherent ultrathin structure is obtained.^[^ ^1a^ ^]^ However, layer‐by‐layer interactions, such as van der Waals forces, exist between the Ti_3_C_2_T*_x_* nanosheets, preventing their subsequent exfoliation and dispersion. Consequently, multilayer Ti_3_C_2_T*_x_* tends to form a stable accordion‐like structure right after the selective etching process.^[^ ^1^, ^10^ ^]^ Although horizontal sliding of the Ti_3_C_2_T*_x_* interlayers occurs sometimes,^[^ ^11^ ^]^ spontaneous exfoliation of the MXene monolayers is difficult. However, multilayered Ti_3_C_2_T*_x_* can be dispersed as a stable colloidal solution after 4 h of ultrasonic treatment using dimethyl sulfoxide as an intercalator.^[^ ^12^ ^]^ In addition, to promote multilayer Ti_3_C_2_T*_x_* delamination, Ghidiu et al. reportedly used Li^+^ ions as intercalators to fill and expand the Ti_3_C_2_T*_x_* layers, and the corresponding exfoliation yield and colloidal concentration were 45% and 2 mg mL^–1^, respectively.^[^ ^2a^ ^]^ In these typical intercalation–exfoliation strategies, the utilization of ultrasound is essential.^[^ ^10a^ ^]^ However, ultrasonic treatment results in the formation of basal‐plane defects and reduces the lateral size of monolayer MXenes, leading to a decrease in the electrical conductivity of these materials.^[^ ^13^ ^]^ Therefore, there is an urgent need to develop delamination methods that lead to high MXene yields and do not require ultrasonic treatment.

**Figure 1:**
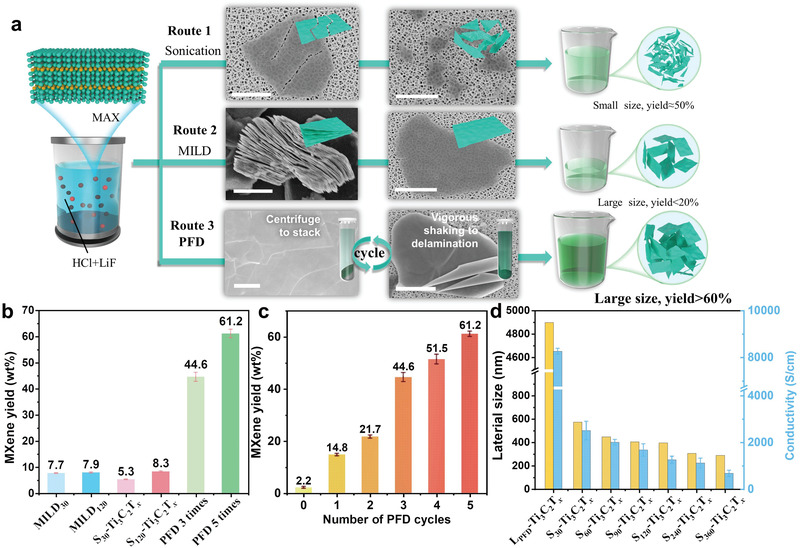
Exfoliation of Ti_3_C_2_T*_x_*: a) Schematic of conventional ultrasonic treatment, MILD, and PFD methods for Ti_3_C_2_T*_x_* exfoliation; scale bar: 2 µm. b) Yield of the dispersed Ti_3_C_2_T*_x_* MXene after different treatments and c) yield of the dispersed Ti_3_C_2_T*_x_* MXene obtained after PFD for different durations. (d) Size and conductivity of the MXenes prepared via ultrasonication performed for different durations.

In 2016, Shahzad et al. developed the minimally intensive layer delamination (MILD) method for the production of MXene flakes, resulting in fewer defects and large flakes (**Figure** **1**a).^[^ ^5^, ^10^ ^]^ In the MILD method, manual shaking is used as an alternative to sonication for the delamination of multilayered MXenes. This method has become the dominant route to prepare MXene materials for studies/applications that require high‐quality large flakes, especially in electronics.^[^ ^6a^ ^]^ In addition, a series of new schemes for the preparation and application of MXenes have been reported, including the intercalation of organic alkali macromolecules,^[^ ^14^ ^]^ evaporated nitrogen,^[^ ^15^ ^]^ electrochemical etching,^[^ ^16^ ^]^ alkali etching,^[^ ^8a^ ^]^ Lewis acid etching,^[^ ^9^ ^]^ and ultrasonic etching.^[^ ^17^ ^]^ However, most of these methods primarily generate multilayer MXene nanosheets, along with a small proportion of isolated single‐layer flakes. Recently, Huang and Wu proposed a novel freeze‐thaw‐based approach for the exfoliation of MXene nanosheets.^[^ ^18^ ^]^ In this method, the volume expansion caused by water‐freezing is used to propel the exfoliation of MXene layers, allowing the separation of large, wrinkled MXene sheets. Using this method, a yield of 39% can be achieved without sonication. We also recently reported an ultrasound‐free method for the spontaneous dispersion of MXene nanosheets in organic solvents.^[^ ^19^ ^]^ Despite these advances, an efficient and rapid delamination protocol is required to facilitate the industrial production of large defect‐free 2D MXenes with high yields.

Thus, with the aim of boosting the yield of large MXene flakes, we propose a power‐focused delamination (PFD) scheme, in which the shear stress is applied on the interface between a fixed MXene precipitate and a vortex fluid. Briefly, the sediment of an etched multilayer MXene was closely stacked and laminated after an intense centrifugation. During the PFD procedure, the impact force generated by the vortex supernatant fluid is concentrated on the surface of the MXene sediment. Thus, using this method, a focused shear can be exerted to exfoliate few‐ to single‐layer MXenes from the surface of an accordion‐like unexfoliated MXene. The yield obtained using this method is significantly higher than that obtained using the MILD method. After five cycles of the PFD, the yield of large defect‐free Ti_3_C_2_T*_x_* MXene nanosheets reached 61.2%, coupled with a colloidal concentration of 20.4 mg mL^–1^ without any sonication. The relatively large lateral dimensions of the MXene sheets allowed an easy fabrication of nanometer‐thin MXene field‐effect transistor (FET) devices, which exhibited electrical conductivities up to 25 000 S cm^‐1^. Our investigation further revealed that the compact precipitates of the multilayer MXenes could effectively absorb the kinetic energy of the flowing water for a rapid exfoliation. Using this strategy, high‐yield large‐sized defect‐free Ti_3_C_2_T*_x_* nanosheets can be readily synthesized and scaled‐up without ultrasonication. The as‐prepared freestanding MXene membranes exhibited an excellent absolute electromagnetic shielding of 35 419 dB cm^2^ g^–1^, transcending those of metals, graphene/carbon nanotubes, and MXene materials prepared through conventional methods. Such high‐quality MXene nanosheets prepared using the PFD scheme are expected to promote the research on large‐scale and size‐dependent MXenes as well as expand the application scope of MXenes.

## 2 Results and Discussion

### 2.1 Preparation of PFD Ti_3_C_2_T*_x_* MXene (L_PFD_‐Ti_3_C_2_T*_x_*)

As shown in Figure 1a, we adopted the LiF + HCl etching strategy, which is one of the most popular techniques, to remove Al from the MAX phase of Ti_3_AlC_2_ and initiate Li^+^ intercalation to weaken the interlayer attraction, which facilitated the subsequent delamination.^[^ ^2a^ ^]^ The LiF+HCl scheme is a relatively safe method, as it does not require the use of concentrated hydrofluoric acid. Moreover, this scheme has more viability for being scaled up to industrial production and application compared to the traditional two‐step method (etching by hydrofluoric acid and intercalation by organic solvents or organic alkalis).^[^ ^12^, ^14^ ^]^ The exfoliation of multilayer MXene nanosheets requires the application of additional forces and energy on the freshly etched Ti_3_C_2_T*_x_*. Ultrasonication is commonly used for the exfoliation of 2D materials, such as graphene, h‐BN, MoS_2_, and so on.^[^ ^20^ ^]^ Notably, the high‐energy‐density microjets and shock waves (≈kW L^‐1^) induced by the ultrasonic waves efficiently exfoliate the 2D material in a dispersant solution as well as cleave the nanosheets and generate basal‐plane defects.^[^ ^21^ ^]^ As shown in Route 1, in the conventional sonication–exfoliation method, Ti_3_C_2_T*_x_* is broken into small pieces, which limits the performance and applicability of 2D MXenes.^[^ ^13^ ^]^ Intriguingly, the shear produced by hand‐shaking or a vortex fluid can also facilitate exfoliation and dispersion of the Ti_3_C_2_T*_x_* MXene (Route 2).^[^ ^5^ ^]^ However, the overall energy density supplied into the system through hand‐shaking or shear mixers for exfoliation is considerably limited (≈100 W L^–1^).^[^ ^21b^ ^]^ Moreover, the multilayer MXene particles and vortex fluid move almost in unison. As expected, only a small fraction of the kinetic energy dissipated in the fluid is directly utilized for exfoliation, and most of the energy is consumed in the synergistic motion of the solution and the multilayer Ti_3_C_2_T*_x_* particles. Consequently, significantly low yields of single‐ to few‐layer MXene nanosheets are obtained during experiments (Movie S1, Supporting Information).^[^ ^5^ ^]^ To overcome the limitations of the hand‐shaking and sonication‐exfoliation methods, we propose the PFD scheme for efficiently preparing ultrathin MXene nanosheets (Route 3). The PFD‐assisted exfoliation method is schematically demonstrated in Figure S1a (Supporting Information). In this method, after the selective etching, the multilayered Ti_3_C_2_T*_x_* is initially centrifuged to form a stable sediment layer at the bottom of the solution (Movie S2, Supporting Information). Afterward, the as‐prepared MXene sediment and solution layer are vigorously oscillated together on a vortex shaker. The shear stress generated by the flow of the supernatant from the oscillation is mainly concentrated on the surface of these multilayer Ti_3_C_2_T*_x_* precipitates. This shear stress allowed the peeling and delamination of the Ti_3_C_2_T*_x_* MXene nanosheets and exposed new Ti_3_C_2_T*_x_* surfaces. Therefore, in this process, the MXene nanosheets would be continuously exfoliated until the precipitate is completely dispersed. In addition, the properties and yields of the Ti_3_C_2_T*_x_* MXenes prepared from different MAX phase precursors often vary.^[^ ^10^, ^22^ ^]^ Therefore, the same batch of the Ti_3_AlC_2_ material was selected to compare the different preparation schemes. Moreover, a commonly used commercial Ti_3_AlC_2_ material, prepared from graphitic carbon rather than the TiC, with a relatively higher yield was used as the raw material in this study. The aim of this study was to identify a general strategy for enhancing the yield and performance of Ti_3_C_2_T*_x_* prepared from commercial raw materials.

The scanning electron microscopy (SEM) image of the intermediates showed the morphologies of each stage, starting from the Ti_3_AlC_2_ precursor to the exfoliated large‐area and ultrathin Ti_3_C_2_T*_x_* nanosheets, during the PFD process (Figures S1–S3, Supporting Information). The lateral size of the Ti_3_C_2_T*_x_* MXene prepared using the traditional sonication–exfoliation method was much smaller than that of the PFD‐synthesized MXene because of the sonication‐induced breakage. Further, the MXene nanosheets fabricated using the MILD and PFD methods displayed similar morphologies and sizes (Figure 1a). However, the commercial applications of MXene are particularly focused on the exfoliation efficiency and production yield of high‐quality MXene nanosheets. Although both sonication and shaking could exfoliate the MXene nanosheets, the resulting yields and final colloidal concentrations were fairly low for both the methods as shown in Figure 1b and Figure S4 (Supporting Information). In contrast, the PFD method remarkably improved the exfoliation efficiency of the Ti_3_C_2_T*_x_* MXenes. After three cycles of the PFD process, the concentration of the colloidal dispersion reached 14.9 mg mL^–1^ (44.6% yield), which is significantly higher than that obtained using the MILD method after 120 min of shaking (2.63 mg mL^–1^ concentration and 7.9% yield). Furthermore, the delamination yield was further enhanced by increasing the number of the PFD cycles, reaching an impressive value of 61.2% after five cycles, as illustrated in Figure 1c. Therefore, these results suggest that separating the MXene sediment phase from the dispersant solution via centrifugation combined with vortex‐induced oscillations results in a more efficient exfoliation and dispersion of the monolayers. Further, we used dynamic light scattering (DLS) and conducted conductivity measurements to evaluate the size distribution and electrical conductivity of the Ti_3_C_2_T*_x_* nanosheets prepared using the PFD and sonication–exfoliation (performed for different durations) methods in order to elucidate the influence of sonication. As expected, the average particle sizes and conductivity of the sonication‐derived layered MXene nanosheets gradually decreased from ≈600 to 300 nm and 667 to 2500 S cm^‐1^, respectively, with the increasing sonication time. In contrast, the MXene nanosheets fabricated using the PFD technique exhibited a much larger average diameter of ≈4900 nm and an electrical conductivity of 8260 ± 130 S cm^‐1^ (film thickness: 11 µm), demonstrating a minimal structural degradation and significantly enhanced performance (Figure 1d and Table S1, Supporting Information and Figures S5–S7, Supporting Information). We compared the electrical conductivity of our MXene materials with those of the reported pure MXene films (Figure S8, Supporting Information and Table S2, Supporting Information). Obviously, the electrical conductivity of the PFD‐synthesized MXene nanosheets was higher than the sonication‐synthesized MXene nanosheets, accentuating the superiority of the PFD strategy.

**Figure 2:**
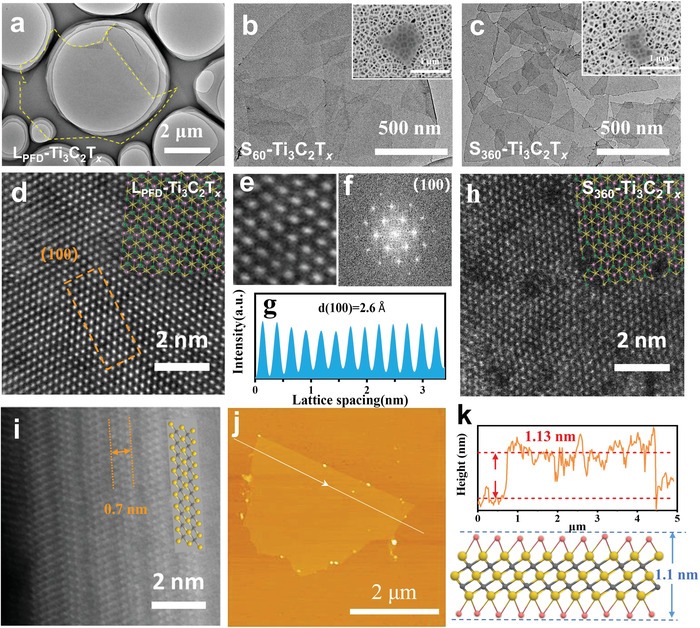
TEM images of a) L_PFD_‐Ti_3_C_2_T*_x_*, b) S_60_‐Ti_3_C_2_T*_x_*, and c) S_360_‐Ti_3_C_2_T*_x_*. d) Atomic‐resolution HAADF‐STEM image of the L_PFD_‐Ti_3_C_2_T*_x_* MXene. e) Enlarged atomic‐resolution HAADF‐STEM image of L_PFD_‐Ti_3_C_2_T*_x_*. f) Fast Fourier transform pattern of L_PFD_‐Ti_3_C_2_T*_x_* MXene. g) Line intensity profile acquired along the orange square in (d). h) Atomic‐resolution HAADF‐STEM image of the S_360_‐Ti_3_C_2_T*_x_* MXene. i) Atomic‐resolution HAADF‐STEM image of a freshly etched Ti_3_C_2_T*_x_* MXene. j,k) AFM image of the monolayer L_PFD_‐Ti_3_C_2_T*_x_* MXene nanosheet loaded on a Si wafer.

Transmission electron microscopy (TEM) measurements confirmed that the MXene nanosheets prepared by the PFD method (L_PFD_‐Ti_3_C_2_T*_x_*, where L represents a large nanosheet) possessed clean surfaces and large lateral sizes (**Figure** **2**a and Figure S9, Supporting Information). To determine the transverse dimensions of the L_PFD_‐Ti_3_C_2_T*_x_* MXene nanosheets, we performed a statistical analysis of more than 280 randomly selected nanosheets, and more than 92.7% of these nanosheets exhibited transverse dimensions of 2.0–9.7 µm (Figures S10 and S11, Supporting Information). In contrast, the TEM images of the sonication–exfoliated MXenes (Figure 2b,c) showed that the size of the Ti_3_C_2_T*_x_* MXene layers apparently reduced after 60 or 360 min of ultrasonication (S_60_‐, S_360_‐Ti_3_C_2_T*_x_*). Furthermore, with an extended sonication time, more scission and shattering of the Ti_3_C_2_T*_x_* nanosheets were observed in the TEM images of S_360_‐Ti_3_C_2_T*_x_*; this result is in agreement with our DLS and conductivity measurement results. The Ti_3_C_2_T*_x_* MXenes prepared by either PFD or the sonication method exhibited transparent structures and clear boundaries. Evidently, the sonication process mainly affected the lateral dimensions of the Ti_3_C_2_T*_x_* MXene nanosheets (Figures S10–S12, Supporting Information). For comparison, the ultralarge Ti_3_C_2_T*_x_* nanosheets in the aqueous suspension were separated by a differential centrifugation method (see methods, Supporting Information). Figure S13 (Supporting Information) shows the relative content of the screened ultralarge Ti_3_C_2_T*_x_* nanosheets in the prepared L_PFD_‐Ti_3_C_2_T*_x_* and S_60_‐Ti_3_C_2_T*_x_* MXene solutions. The proportion of the large MXene nanosheets in the L_PFD_‐Ti_3_C_2_T*_x_* MXene solution was 6.1 times higher than that in S_60_‐Ti_3_C_2_T*_x_*, indicating that the PFD strategy can be applied to realize both exfoliated MXenes with high yields and large sheet sizes. The N_2_ adsorption–desorption isotherms were recorded to assess the textural properties of the L_PFD_‐Ti_3_C_2_T*_x_* and S_60_‐Ti_3_C_2_T*_x_* MXene. As shown in Figure S14 (Supporting Information), both L_PFD_‐Ti_3_C_2_T*_x_* and S_60_‐Ti_3_C_2_T*_x_* MXene exhibit type II isotherms, indicating that MXene materials do not possess mesoporous structure. Meanwhile, the surface area of L_PFD_‐Ti_3_C_2_T*_x_* (12.5 m^2^ g^‐1^) is slightly higher than that of S_60_‐Ti_3_C_2_T*_x_* (10.8 m^2^ g^‐1^), which may be due to the compact stacking of MXene materials with smaller lateral size during the freeze‐drying process.^[^ ^23^ ^]^ The XPS survey spectra of L_PFD_‐Ti_3_C_2_T*_x_* and S_60_‐Ti_3_C_2_T*_x_* show that the chemical compositions of MXenes prepared by both strategies are composed of C, Ti, O, and F.^[^ ^24^ ^]^ The chemical states of surface elements can be obtained by deconvolution of Ti 2p, C 1s, F 1s and O 1s signals, as shown in Figure S15 (Supporting Information).^[^ ^24^, ^25^ ^]^ It is worth noting that the content of TiO_2_ species in the S_60_‐Ti_3_C_2_T*_x_* material prepared by the ultrasonic strategy is significantly higher than that of the L_PFD_‐Ti_3_C_2_T*_x_* material (Figure S16, Supporting Information). This phenomenon is due to the interaction of water and a small amount of dissolved oxygen with the MXene material during the sonication process.^[^ ^26^ ^]^ The Ti_3_C_2_T*_x_* MXene nanosheets with large area are more favorable for long‐term storage in air, which has been studied in detail.^[^ ^27^ ^]^ The content of TiO_2_ species is positively correlated with the degree of oxidation for Ti_3_C_2_T*_x_* MXenes.^[^ ^25a^ ^]^ The L_PFD_‐Ti_3_C_2_T*_x_* and S_60_‐Ti_3_C_2_T*_x_* MXene materials, freshly prepared and stored in air for 7 d, were further explored by XPS spectroscopy and Raman spectroscopy. For the L_PFD_‐Ti_3_C_2_T*_x_* MXene with larger lateral size, both the XPS and Raman spectra showed that one week of environmental exposure did not lead to an increase in the content of TiO_2_ species in the material (Figure S17, Supporting Information). Conversely, the XPS spectra of S_60_‐Ti_3_C_2_T*_x_* material stored under ambient conditions for 7 d showed that the signal of TiO_2_ species was significantly elevated compared to the freshly prepared material. The Raman spectra of S_60_‐Ti_3_C_2_T*_x_* MXene materials also showed an additional peak of anatase TiO_2_ in the region of 150 cm^–1^ after one week of ambient exposure. And the two broad peaks between 1200 and 1600 cm^–1^ are strongly enhanced, characteristic of the D and G modes of graphitic carbon (Figures S18 and S19, Supporting Information).^[^ ^24^, ^28^ ^]^ This is due to the degradation of small sheets of MXene upon exposure to the environment. These results suggest that the larger area of the L_PFD_‐Ti_3_C_2_T*_x_* MXene nanosheets is beneficial for long‐term storage, consistent with previous studies.^[^ ^27^ ^]^

The atomic‐resolution 2D structure of the Ti_3_C_2_T*_x_* MXenes was clearly visualized using high‐angle annular dark‐field scanning transmission electron microscopy (HAADF‐STEM). In the HAADF‐STEM images (Figure 2d), the orderly arrangement of the three Ti atomic layers appeared brighter than the overlapped C atoms owing to the intrinsic *Z*‐contrast. The typical hexagonal symmetry of the Ti‐atom alignment on the L_PFD_‐Ti_3_C_2_T*_x_* MXene surface is further illustrated in Figure 2d. The crystal structure of L_PFD_‐Ti_3_C_2_T*_x_* was determined by atomic superposition, and the corresponding fast Fourier transform (FFT) patterns are presented in Figure 2e,f. These FFT patterns reveal that L_PFD_‐Ti_3_C_2_T*_x_* exhibits a diffraction pattern characteristic of the (100) crystal plane. Additionally, the integrated pixel intensity of the (100) crystal plane of Ti_3_C_2_T*_x_* is shown in Figure 2g, where the average crystal plane spacing is 0.26 nm. Notably, the atomic‐resolution HAADF‐STEM images presented in Figure 2h show that the monolayer L_PFD_‐Ti_3_C_2_T*_x_* MXene contained considerably fewer basal‐plane defects compared to the monolayer Ti_3_C_2_T*_x_* MXene prepared by the conventional sonication method. This indicates that the ultrasonic treatment also tends to cause loss of surface Ti atoms from the MXene surface, leading to the formation of numerous atomic defects, possibly because of the local intense heating and pressure shock caused by the ultrasonic waves. Figure 2i shows a cross‐sectional HAADF image of a freshly etched Ti_3_C_2_T*_x_* before delamination, and an Ti_3_C_2_ monolayer with thickness of 0.7 nm is explicitly observed. The thickness of the L_PFD_‐Ti_3_C_2_T*_x_* MXene was determined to be ≈1.13 nm through atomic force microscopy (AFM) measurements. The L_PFD_‐Ti_3_C_2_T*_x_* was only slightly thicker than the monolayer Ti_3_C_2_ because of the existence of T*_x_* groups and H_2_O molecule coverage, which occurred when the Ti_3_C_2_ layer was exposed after the delamination (Figure 2j,k). The negative electrostatic charges on the hydrophilic L_PFD_‐Ti_3_C_2_T*_x_* nanosheets are considered to stabilize the aqueous solution predominantly composed of single‐layered flakes. The thickness and number of layers of L_PFD_‐Ti_3_C_2_T*_x_* MXene were analyzed through more AFM and TEM images. AFM analysis showed that among the 27 L_PFD_‐Ti_3_C_2_T*_x_* flakes for which thicknesses could be measured, more than 80% of them were 1–2 nm thick ( Figure S20, Supporting Information). However, except for the wrinkled MXene nanosheets, it is difficult to directly confirm the number of layers of MXene through AFM images. High‐resolution TEM analysis is a more convenient method for identifying the number of layers in MXene materials (Figure S21, Supporting Information). The analysis of 124 flakes suggested that 81.1% of the flakes were 1‐2 layers thick (Figure S22, Supporting Information). The ratio of monolayer and bilayer in L_PFD_‐Ti_3_C_2_T*_x_* MXene is higher than that in the MXene material prepared by sonication–exfoliation method^.[^ ^2a^ ^]^ Considering the effect of restacking and folding of MXene nanosheets in the sample preparation step before thickness analysis, the actual monolayer ratio should be higher. Thus, the PFD strategy can easily produce MXene flakes with high yield, large‐area and excellent performance.

### 2.2 High Conductivity of the Individual L_PFD_‐Ti_3_C_2_T*_x_* MXene Nanosheets

**Figure 3:**
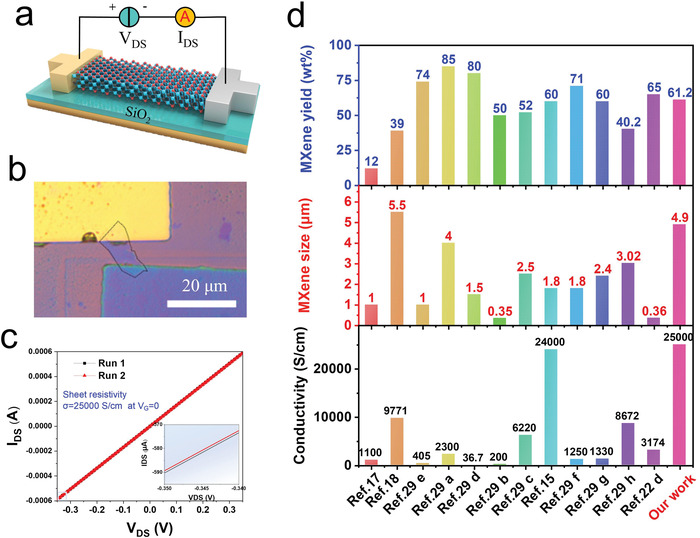
Conductivity measurements and comparison of properties of the as‐prepared L_PFD_‐Ti_3_C_2_T*_x_* MXenes. a) Schematic of the conductivity testing scheme of the L_PFD_‐Ti_3_C_2_T*_x_* MXene nanosheets. b) Optical microscope image of the FET device with L_PFD_‐Ti_3_C_2_T*_x_* MXene nanosheets. c) *I*_DS_–*V*_DS_ curve for the device with *V*_G_ = 0. The inset is a schematic of the error obtained in the two tests at the maximum voltage; the difference is almost negligible and can be considered as the test error. d) Comparison of the reported conductivity, size, and material yield of the Ti_3_C_2_T*_x_* MXene materials prepared by the PFD process.

**Figure** **3**a shows a schematic of the setup used for measuring the electrical conductivity of an L_PFD_‐Ti_3_C_2_T*_x_* MXene nanosheet, having a thickness of 1.8 nm (monolayer or bilayer), on a silicon wafer substrate with an oxide layer. Due to the relatively large size of the individual L_PFD_‐Ti_3_C_2_T*_x_* nanosheets, we could directly observe the MXene sheets through an optical microscope and fabricate the FET device (Figure S23, Supporting Information). The two terminals of the nanometer‐thin MXene flakes were easily connected to an Al electrode (drain) and a Pt electrode (source) with manipulations under an optical microscope. The device was then connected to an external circuit to measure and record the voltage and current signals at zero gate voltage (*V*_G_ = 0). The large high‐quality MXene flakes reduce the operational difficulties associated with electrode deposition and the subsequent measurement of electronic properties of the bridging ultrathin layers (Figure 3b). In Figure 3c, the current–voltage (*I*_DS_–*V*_DS_) curve of drain–source exhibits a strong linear correlation, and the device hardly changed over multiple cycles, indicating a stable ohmic resistance of the L_PFD_‐Ti_3_C_2_T*_x_* MXenes. The average resistivity of the device was 39.8 µΩ m, and the conductivity of the L_PFD_‐Ti_3_C_2_T*_x_* MXene was calculated as 25 000 S cm^‐1^ (see Supporting Information for calculation details), which is slightly higher than that reported for the Ti_3_C_2_T*_x_* MXenes.^[^ ^15^ ^]^ The improved electronic properties can be attributed to the high‐integrity structure and fewer defects in the monolayer L_PFD_‐Ti_3_C_2_T*_x_* MXenes. In addition, a single‐flake device eliminates the conductivity loss caused by the electronic transitions between stacked MXene sheets in layered films. Thus, single‐flake devices are more favorable for obtaining high conductivities.

The yields, lateral dimensions, and electrical conductivities of the prepared mono/few‐layer Ti_3_C_2_T*_x_* materials were compared to quantify the advantages of various preparation strategies. As shown in Figure 3d, our PFD strategy can facilitate the fabrication of MXenes with high yields, large lateral sizes, outstanding electrical conductivities (Tables S2 and S3, Supporting Information).^[^ ^15^, ^17^, ^18^, ^22^, ^29^ ^]^ Therefore, the PFD strategy is excellent for the production of mono/few‐layer Ti_3_C_2_T*_x_* materials with both high yields and high performances.

### 2.3 Mechanistic Analysis

**Figure 4:**
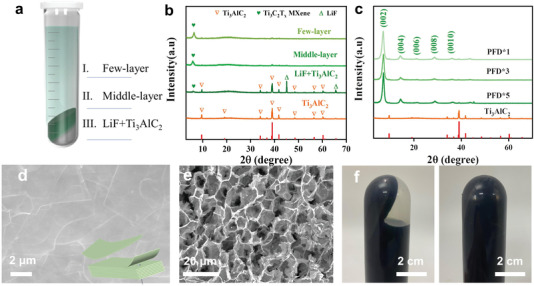
Evidence of MXene precipitation stratification: a) Schematic of the solid and supernatant after centrifugation. b) XRD patterns of the different components obtained by centrifugal precipitation. c) XRD patterns of the MXenes obtained after different numbers of PFD cycles. SEM images of d) the multilayer Ti_3_C_2_T*_x_* MXene precipitate and e) the intermediate state of the Ti_3_C_2_T*_x_* MXene during the PFD process. f) Photographs of the solid and supernatant MXene in an inverted centrifuge tube before and after five PFD cycles.

**Figure 5:**
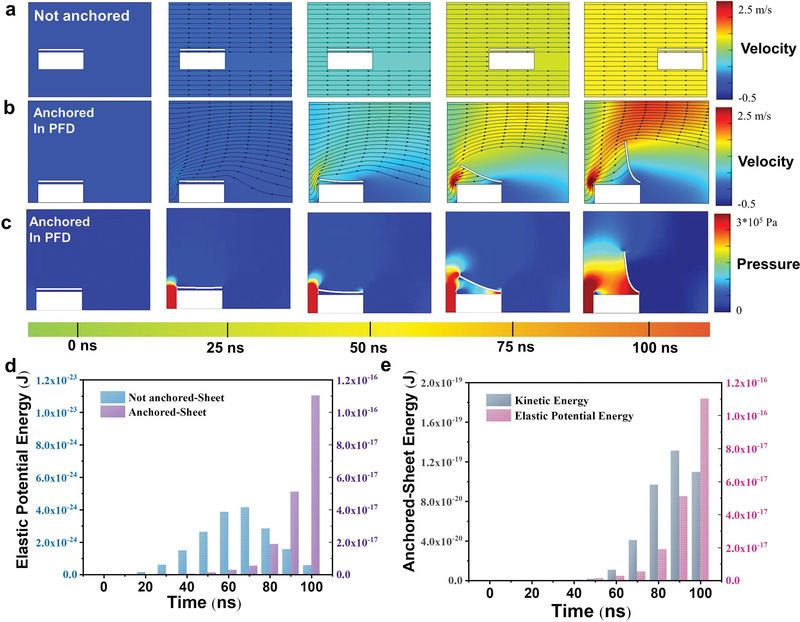
Hydrodynamic simulation of solution–exfoliation: Variation in water flow and block motion when the block is a) not anchored during conventional shaking and b) anchored in the PFD method with changes in the velocity of water flow and c) pressure. d) Elastic potential energy acquired by the surface nanosheet during the delamination process under the not anchored and anchored PFD conditions. e) Kinetic and elastic potential energy acquired by the surface layer during the PFD.

Two key experimental details should be noted: 1) after the washing, redispersion of the multilayer Ti_3_C_2_T*_x_* MXene precipitate is difficult, and vigorous shaking for a long time is required to achieve dispersion. We noticed that a more difficult redispersion process resulted in a higher yield of the Ti_3_C_2_T*_x_* nanosheets. 2) The precipitate contained multiple components, and with the increasing number of PFD cycles, the precipitate was gradually divided into three parts (**Figure** **4**a and Figures S22 and S23, Supporting Information). X‐ray diffractometry (XRD) analysis revealed that the top and middle layers were the Ti_3_C_2_T*_x_* MXenes (Figure 4b), whereas the bottom layer was a mixture of impurities and incompletely etched raw Ti_3_AlC_2_. In addition, as the number of PFD cycle increased, the volumes of the upper and middle layers in the precipitate initially increased and then decreased, which correspond to interlayer expansion and separation (Figure S24, Supporting Information). Eventually, most of the Ti_3_C_2_T*_x_* MXenes were transferred from the sediment to the upper liquid layer in the form of single‐ to few‐layer MXenes. The XRD patterns revealed that the number of PFD cycle does not affect the crystal structure of the upper Ti_3_C_2_T*_x_* MXene layer, but only increases the concentration of the Ti_3_C_2_T*_x_* MXenes in the colloidal solution (**Figure** **5**c). This indicates that Ti_3_C_2_T*_x_* MXene is released from the precipitate to the solution during the PFD process. The SEM images of the different regions of the precipitate (Figures S25 and S26, Supporting Information and Table S4, Supporting Information) demonstrated that the surface layer comprised of few‐layer MXenes that were approaching dispersion into the colloid, suggesting that this is the main region of the PFD action. After the initial centrifugation, the multilayers and few‐layer Ti_3_C_2_T*_x_* MXenes were firmly stacked at the interface between the sediment and the supernatant layer (Figure 4d). The subsequent vigorous oscillation during the PFD process delivered a strong vortex flow on these tightly stacked MXene nanosheets. The intermediate state during the oscillation exhibited a much coarser surface that consisted of small tilted Ti_3_C_2_T*_x_* MXene flakes exposed into liquid layer (Figure 4e). The viscosities of the upper and middle liquid layers also increased significantly after the PFD cycles because of the elevated MXene concentration in the aqueous solution (Figure 4f).

### 2.4 Hydrodynamic Simulation

The superior exfoliation efficiency of the PFD process can be attributed to the interactions between the compacted sediment of the multilayer MXenes and the flow of the supernatant during the oscillations. To further unravel the mechanism of the PFD exfoliation and understand the experimentally observed phenomenon in relation to the effects of the oscillation‐induced flow during the redispersion after the centrifugation, we performed a computational fluid dynamics (CFD) simulation of the MXenes. As shown in Figure 5, the simplified model consists of three parts: a fluid, monolayer nanosheet, and bottom supporting block as the unexfoliated MXene substrate. In the simulations, we focused on the delamination of a single nanosheet from the multilayer phase (bulk phase) and the energy transition between the kinetic energy of the flow and the elastic potential energy of the MXene (see the modeling and simulation details). This is because the latter represents the deformation of the MXene nanosheet during the exfoliation process, and is critical for the mechanical delamination. However, this model does not consider thermal and electrostatic effects and collision between the particles. Despite these limitations, it is still a good analytical model for this system, because elastic deformation is the most important factor affecting the exfoliation of the nanosheets during the MILD and PFD processes. Our experimental results indicated that, in the PFD method, the kinetic energy of the water flow is efficiently utilized, which is further supported by the CFD simulations. In the conventional manual shaking or oscillation without the formation of sediment by centrifugation, the water and MXene particles move synchronously, resulting in only a slight interaction between the fluid and the solid phases. Although the gradual increase in water velocity occasionally causes an uneven shear on the MXene blocks, the dispersed MXene and the fluid quickly reach a synergistic motion, and force is exerted uniformly (Figure 5a). Therefore, the impact of the water flow is mainly converted into the kinetic energy of the floated MXene particles, and the interconversion of kinetic energy does not increase the elastic potential energy of the surface MXene nanosheet, and thus, does not result in an effective delamination. In contrast, a significant flux concentration appears in the vicinity of the surface layer, when the supernatant fluid starts to flow on the firmly anchored MXene block, implying that a high shear rate is generated on the superficial nanosheet (Figure 5b). Moreover, the pressure of the water flow at the gap between the surface sheet layer and the block, with an anchored bottom, increases drastically (Figure 5c). Simultaneously, the high shear rate and pressure difference of the water flow can easily deform the nanosheets and result in the delamination of the MXene nanosheets as demonstrated in our simulation.

Energy is required for the elastic deformation and to overcome other forces between the nanosheets, and this required energy is provided by the kinetic energy of the water flow. Therefore, we modeled the kinetic and elastic potential energies of the blocks and surface nanosheets under different conditions. In the model of the conventional shaking with a free block, the kinetic energies of the surface nanosheet and bottom block increase simultaneously (Figure S27, Supporting Information), suggesting that the two components move in coherently, as shown in Movie S3 (Supporting Information); thus, no effective delamination of the nanosheets occurs. For the MXene nanosheets in the surface region of the precipitate, the kinetic energy gained during the PFD process is much lower than that in the free‐block condition (Figure S28, Supporting Information). This is presumably because most of the impact force from the water flow is transformed into the elastic potential energy in the nanosheets. Figure 5b and Figure S28 (Supporting Information) show that the kinetic energy of the water is more focused on the surface layer in the PFD method than in the conventional oscillation or manual shaking processes. Furthermore, the energy utilization efficiency of the bottom‐anchored stress‐concentrated scheme is seven orders of magnitude greater than that of the free‐block case analyzed through simulations (Figure 5d). Thus, the PFD method can more effectively transform the impact force of the water flow into elastic potential energy, rather than kinetic energy, of the unexfoliated MXene particles (more than 99% of the energy is converted to surface strain, as shown in Figure 5e). This suggests that the PFD method is much more effective in delaminating MXenes, which is consistent with our experimental observations.

### 2.5 Electromagnetic Shielding

**Figure 6:**
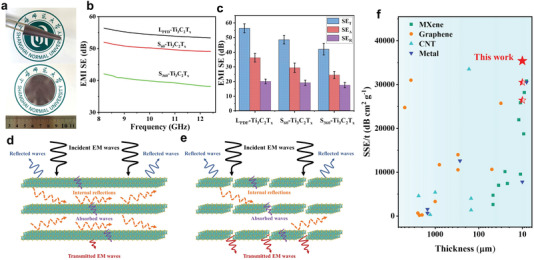
Electromagnetic shielding performance of the Ti_3_C_2_T*_x_* MXenes. a) Photographs of the as‐prepared L_PFD_‐Ti_3_C_2_T*_x_* film for the electromagnetic shielding test. b) SE, c) total SE (SE_T_), and absorption (SE_A_) and reflection (SE_R_) contributions of the L_PFD_‐Ti_3_C_2_T*_x_*, S_60_‐Ti_3_C_2_T*_x_* and S_360_‐Ti_3_C_2_T*_x_* MXene films. Schematic of the electromagnetic shielding of d) L_PFD_‐Ti_3_C_2_T*_x_* prepared by the PFD method and e) S_360_‐Ti_3_C_2_T*_x_* MXene films prepared by the conventional sonication–exfoliation method. f) Comparison of the SSE/*t* values of our films with those reported in the literature.

Typically, for a single‐component material, the electromagnetic interference shielding effectiveness value is proportional to the conductivity and thickness of the film. Therefore, we conducted electromagnetic shielding tests on the L_PFD_‐Ti_3_C_2_T*_x_* or Ti_3_C_2_T*_x_* films prepared under different sonication times; for the tests, films with same masses and thicknesses were used (**Figure** **6**a). As shown in Figure 6b, the L_PFD_‐Ti_3_C_2_T*_x_* MXene film showed an excellent shielding performance. To demonstrate the difference in the electromagnetic shielding (SE) efficiency of the samples prepared under different sonication times, the specific reflection (SE_R_) and absorption (SE_A_) contributions were calculated, as illustrated in Figure 6c. Obviously, the effect of the differences in the layer sizes was negligible on the SE_R_. However, the films prepared from larger monolayer Ti_3_C_2_T*_x_* nanosheets demonstrated excellent SE_A_ values. This is because the reflection in this case is mainly caused by the mismatch between the interfacial impedance of the incident electromagnetic waves and the electromagnetic shielding material, whereas the absorption is mainly determined by the characteristics of the shielding material. When an electromagnetic radiation is incident on the nanosheet, a fraction of the incident waves is immediately reflected at the surface, whereas the remaining waves are transmitted through the MXene lattice structure. The interaction of the electromagnetic waves with the MXene, as they propagate through the nanosheets, induces currents, resulting in ohmic losses, and the electromagnetic waves inside the material are reflected multiple times between the interfaces. The multiple reflection effect is included in the absorption contribution, and these interlayer re‐reflected waves are absorbed or dissipated in the material as heat. Eventually, the energy of the electromagnetic waves decreases, and the remaining unabsorbed electromagnetic waves pass through the shielding material (Figure 6d,e). In general, the conductivity of Ti_3_C_2_T*_x_* is usually influenced by the nanosheet size, surface groups, defect concentration, and contact resistance between the nanosheets. Notably, the non‐eliminable contact resistance in the gaps between the monolayer Ti_3_C_2_T*_x_* sheets affects the conductivity between the sheets. However, the large monolayers of Ti_3_C_2_T*_x_* prepared by the PFD method had fewer basal‐plane defects, resulting in more electric dipoles that improved the dielectric loss capability of the material. Moreover, the films prepared from the large monolayers of Ti_3_C_2_T*_x_* also had fewer internal gaps; consequently, the films exhibited a stronger electromagnetic shielding ability. For the evaluation of the electromagnetic shielding performance of the different component materials, the shielding effectiveness per unit volume (SSE/*t*) value is a more objective criterion. Therefore, we compared the SSE*/t* values of the films derived from monolayer Ti_3_C_2_T*_x_*, with different sizes, with those reported in the literature, as shown in Figure 6f and Table S5 (Supporting Information).^[^ ^5^, ^30^ ^]^ The MXene films showed an outstanding electromagnetic shielding ability compared with those of other carbon‐based and metal‐based shielding materials, and the SSE*/t* values of the Ti_3_C_2_T*_x_* films prepared by the PFD method reached 35 419 dB cm^2^ g^–1^. This result demonstrates the potential of the PFD strategy for electromagnetic shielding and related applications.

## 3 Conclusions

Here, we report a fast and efficient PFD strategy to assist in the preparation of Ti_3_C_2_T*_x_* MXene materials. Through controlled experiments, we demonstrated that in the PFD process, the shear of the vortex fluid, generated through oscillations, is concentrated on the surface layer of the Ti_3_C_2_T*_x_* MXene precipitates. This promotes uncoordinated movement between the surface Ti_3_C_2_T*_x_* MXene nanosheets and the block. This scheme greatly improves the exfoliation efficiency and increases the yield by 6.4 times compared to the sonication–exfoliation and MILD methods. Additionally, the MXene layers prepared by the PFD process have large lateral dimensions, and the morphology damage and loss of properties observed when using the sonication–exfoliation method are prevented. Finally, the SSE*/t* value of the Ti_3_C_2_T*_x_* MXene material prepared via the PFD method is much higher (35 419 dB cm^2^ g^–1^) than those prepared via ultrasonic treatments. In conclusion, this new assisted exfoliation strategy significantly improved the exfoliation efficiency of large‐area monolayer Ti_3_C_2_T*_x_* MXene materials and is expected to open new avenues for varied applications of Ti_3_C_2_T*_x_* MXene materials.

## 4 Experimental Section

### PFD‐Assisted Synthesis of Ti_3_C_2_T*_x_*

The etching reaction in PFD is exactly the same as the etching process used in the MILD process, but the washing and delamination processes are different from those of the MILD method. First, the MXene was washed with 1 m hydrochloric acid, followed by washing with deionized water until neutral pH is achieved. Subsequently, the washing was continued until the MXene underwent spontaneous delamination. The neutral dispersion was oscillated on a vortex shaker (Heidolph Multi Reax oscillator; at 2000 rpm) for 10 min to redisperse the centrifuged precipitate uniformly, and then, the dispersion was centrifuged at 10 000 rpm for 5 min. Then, the shaking and centrifugation steps were repeated five times, and the resulting dispersion was centrifuged at 3000 rpm for 15 min to remove the upper layer of the black colloidal solution, labeled as L_PFD_‐Ti_3_C_2_T*_x_*. The redispersion process may be slow but can be aided by a light stirring using a fine rod. In addition, the volume of the solution should not exceed 2/3 of the centrifuge tube during the shaking.

### Conductivity Measurement of the Monolayer Ti_3_C_2_T*_x_* MXenes

First, a diluted Ti_3_C_2_T*_x_* MXene solution was coated on a plasma‐treated silicon wafer substrate with an oxide layer. Water was removed by heating in a 60 °C vacuum oven. The monolayer Ti_3_C_2_T*_x_* MXene flakes were identified using light microscopy and AFM, the thickness of the flakes was confirmed to be about 1.8 nm. Subsequently, the photoresist (S1818) was spin‐coated onto the silicon wafer substrate (5000 rpm for 45 s) prebaked at 100 °C for 1 min. A mask aligner was used to align the designed shape with one end of the MXene and expose it with a mask. Then, the samples were developed, cleaned with a developer and deionized water (for 60 s in each case), and then baked at 90 °C for 1 min. Next, a 50 nm thick Pt electrode layer was sputtered, and the excess photoresist and metal layers were removed by soaking with acetone. The photoresist was then spun again, and the samples were reprocessed with a mask aligner, developer, and deionized water. After sputtering a 50 nm thick Al layer, the excess photoresist and metal layer were removed by soaking in acetone to complete the electrode construction of single MXene ends. The electrodes were externally connected using silver paste and conductive copper wires, and the electrical properties were measured using a Keithley 4200 semiconductor analyzer.

### Modeling and Simulation Details

To simulate the exfoliation process of the MXene materials in an aqueous solution, a fluid–structure interaction simulation scheme was used to bridge the huge time‐ and length‐scale gaps between the fluid and the multilayered MXene particles. The COMSOL CFD Laminar Flow module and the Solid Mechanics module were used to simulate the fluid–structure interactions that resulted in the exfoliation of the multilayer MXenes. Here, the fluid is modeled by the Navier–Stokes equation, whereas the MXene material is described by a linear elastic material. Figure 5a indicates the region of flow map (blue) and the solid region (white). The right boundary of the fluid region is the inlet flow velocity, and the part of the boundary in the left is the outlet pressure (gauge pressure is 0 Pa). The initial flow velocity is 0 m s^‐1^, and then it increases to 1.4 m s^‐1^ within 50 ns according to a step function. In the current study, the inlet flow velocity was assumed to be consistent. In addition, part of the left boundary is a slip wall with a length of 43 nm, located at a distance of 32 nm from the bottom. The solid region moves freely, and the upper and lower rectangles are constrained by the attachment of the leftmost local boundary, which enabled the upper and lower solid regions to stick together.

The calculations were performed considering a normal temperature of 25 °C. In the fluid part, the mass density of the fluid was defined as 1 g cm^‐3^, which is close to the properties of water at room temperature, and the fluid viscosity was 0.001 Pa s. In the solid section, the density of MXene was defined as 2.9 g cm^‐3^, and its Poisson's ratio was 0.316. Considering the complexity of the multiple MXene particles and multilayered MXene interactions, the model parameters were optimized for computational convenience. The monolayer MXene was 50 nm long and 2 nm thick (overall size), and the multilayer MXene structure was optimized for modeling the interactions between the monolayer and bulk MXenes (the thickness of multilayer MXene is defined as 50 nm). Moreover, the spacing between the monolayer MXene and the underlying bulk MXene was 2 nm. To analyze the effect of bottom anchoring, the left particle of the single‐layer MXene was anchored with the bottom bulk to simulate the unpeeled part at the back of the large‐scale MXene sheet. Due to the limitations of the hydrodynamic simulation system, the 50 nm × 1000 nm (length × width) nanosheets were selected under the consideration of a monolayer thickness of 2 nm. Although this horizontal size is significantly different from the actual size, it is an appropriate choice considering the complexity and representativeness of the calculation.

The mesh numbers for this fluid–structure interaction simulation system were 2369 (liquid) and 316 (solid), respectively. Both the bottom‐anchored and bottom‐unanchored modes of the system operation were performed, and two mesh sensitivity tests were conducted to ensure that the results were independent of the number of elements. In both the computational domains, the sizes of uniform MXene materials were applied, demonstrating the dynamic evolution of fluids and solids within a certain time (240 ns). The model mesh was affected by the displacement and phase change of the solid due to fluid scouring. Therefore, the Moving Mesh interface function was used in the model to incorporate the mesh deformation during the deformation of the moving solid. When the mesh is deformed to a certain extent, the quality of the mesh is greatly reduced, resulting in non‐convergence. Therefore, the automatic remeshing setting was added to distort the mesh by more than 2%, i.e., start the remeshing operation until the final solution is obtained.

The complex interaction between the monolayer MXene material and the underlying bulk material was optimized as the energy barrier for elastic deformation of the MXene material. When the solution starts the shearing motion, the MXene material overcomes the elastic potential energy and exfoliates. The kinetic and elastic potential energy fractions obtained for the MXene monolayers and bulk materials in the fluids were numerically calculated using CFD tools. The entire calculation process was carried out with the COMSOL software (software version).

### Statistical Analysis

The following steps were performed for data preprocessing. Before the test, the sample quality was checked to obtain reliable raw data. OriginPro 2020 software was used for the statistical analysis and graphical representation of the data. Three independent sets of experiments were performed, and the results were obtained in the form of mean ± standard error. Two‐tailed unpaired Student's *t*‐tests were performed to examine the concentration, yield, conductivity, and electromagnetic shielding properties of the MXene materials (*p* < 0.05 was considered statistically significant). The TEM images were analyzed using the Gatan Digital analysis software to determine the layer thickness, and the SEM images were analyzed using the Nano Measurer software to determine the lateral dimensions of the nanosheets. The sample size (*n*) for each statistical analysis is mentioned in the TEM, SEM, and AFM analysis.

## Conflict of Interest

The authors declare no conflict of interest.

## Supporting information

Supporting Information

Supplemental Movie 1

Supplemental Movie 2

Supplemental Movie 3

## Data Availability

The data that support the findings of this study are available in the supplementary material of this article.
