# Supplementary material for: Synthesis of Large‐Area MXenes with High Yields through Power‐Focused Delamination Utilizing Vortex Kinetic Energy
Source: Adv Sci (Weinh). 2022 Aug 17;9(28):2202748. doi: 10.1002/advs.202202748 (PMC9534978; doi:10.1002/advs.202202748)
Supplement: Supplementary file 1 — Supporting Information [file ADVS-9-2202748-s001.pdf]

Supplementary Information for

Synthesis of Large-Area MXenes with High Yields through Power-Focused Delamination Utilizing Vortex Kinetic Energy

Qingxiao Zhang, Runze Fan, Weihua Cheng, Peiyi Ji, Jie Sheng, Qingliang Liao, Huirong Lai, Xueli Fu, Chenhao Zhang, Hui Li**

Shanghai Key Laboratory of Rare Earth Functional Materials and Education Ministry Key Laboratory of Resource Chemistry, Shanghai Normal University, Shanghai 200234, P. R. China

E-mail: chenhao.zhang@shnu.edu.cn; lihui@shnu.edu.cn

Supplementary Methods

1. Chemicals

Ti_3AlC_2 (200 mesh, 99%) was purchased from Laizhou KaiKai Ceramic Materials Co., Ltd. Lithium fluoride (99%) and concentrated hydrochloric acid (36-38%) were purchased from Shanghai Aladdin Biochemical Technology Co., Ltd. The rest of the chemicals used in this experiment were commercial raw materials and were not treated in any way.

2. Material preparation

2.1 Synthesis of Multilayer $Ti_3C_2T_x$

The choice of precursors for MXene is important for the synthesis. Based on previous studies, the differences in the synthesis conditions of Ti_3AlC_2 lead to the formation of impurity phases and densities in Ti_3AlC_2.[1] Therefore, in this study, we used only the same batch of Ti_3AlC_2 material. Etching process: First, 60 mL of 9 M hydrochloric acid was added to the Teflon reactor, then 4.8 g LiF powder was added and stirred for 5 min. Then, slowly add 3.0 g Ti_3AlC_2 in a fume hood and close the lid with a vent when finished, this step will take 10 minutes. Finally, the reaction was carried out at 450 rpm and 45 °C water bath for 24 h. It should be noted that a small slit or vent should be reserved in the Teflon reactor for the release of H_2 generated during the reaction. Also, the mixture of hydrochloric acid and LiF is as hazardous to humans as low concentrations of hydrofluoric acid, so proper protection is necessary.

Washing process: The solution was divided equally in a centrifuge tube after the reaction, and the supernatant was poured off at the end of centrifugation. The precipitate was first acid washed twice with 1 M hydrochloric acid, and then washed with deionized water until the pH value was greater than 6. The acid washing step is

used to wash off the excess LiF more easily. Centrifugation speed in the washing step is 10,000 rpm and the centrifuged precipitate should be well dispersed in each wash.

2.2 Synthesis of S_T-$Ti_3C_2T_x$

Delamination process: Add 100 mL DI water to the washed sediment, then sonicated for different time. Finally, the dispersion was centrifuged at 3500 rpm for 20 min to take the upper black colloidal solution, labeled as S_T-$Ti_3C_2T_x$, here, S represents sonication, and T represents the time of ultrasound. It should be noted that the sonication process was carried out under the protection of an ice-water bath and flowing argon gas.

2.2 Screening of ultra-large $Ti_3C_2T_x$ nanosheets

Ultra-large $Ti_3C_2T_x$ MXene were screened by a differential centrifugation strategy. Directly, the prepared L_{PFD}-$Ti_3C_2T_x$ MXene solution and S_{60}-$Ti_3C_2T_x$ MXene solution were centrifuged at 5000 rpm for 30 min, and the precipitate was collected. Deionized water was then added to the precipitate and shaken to redisperse it. The two samples were designated L_{PFD}-$Ti_3C_2T_x$-select and S_{60}-$Ti_3C_2T_x$-select, respectively, and their solids content was determined by freeze-drying.

3. Test characterization

The crystalline structure was investigated by the X-ray diffraction (XRD; igaku ltimate IV powder X-ray Cu Kα radiation diffractometer). Dynamic light scattering (DLS) and ζ-potential measurements were recorded on a Zetasizer Nanoseries instrument (Mastersize ZS100). The material shapes and morphologies were analyzed by the transmission electron microscopy (TEM, FEI TF20) at an acceleration voltage of 200 kV. Atomic force microscopy (AFM) images was acquired by means of a (AIST-NT, Smart SPM). Scanning electron microscopy (SEM) images were performed on a field-emission HITACHI, S-4800 microscope. The electrical

conductivity of MXene member was performed on a four-point probe system (Guangzhou Four Probes Tech. Co. Ltd., RTS-9). HAADF-STEM were performed on a FEI Titan Themis with a probe corrector at 300 kV. A static mechanical tester (HY-0350, Shanghai Hengyi Precision Instrument Co., Ltd.) was used to test the tensile mechanical properties of the samples, the tensile plastic was 100 mm·min^{-1}, and the sample size was 0.5 cm*3 cm. X-ray photoelectron spectroscopy (XPS) was performed on an X-ray photoelectron spectrometer (Thermo fisher Scientific, K-Alpha, USA) apparatus using an Al Kα X-ray source to investigate their surface electronic states. Raman spectra of the L_{PFD}-$Ti_3C_2T_x$ MXene and S_{60}-$Ti_3C_2T_x$ MXene were obtained using a Raman spectroscope (Horiba Scientific LabRAM HR Evolution, Japan) equipped with a 532 nm laser source. The N_2 adsorption–desorption isotherms were recorded on a Micromeritics ASAP 2460 analyzer at 77.3 K and the surface area was calculated by BET methods.

4. Conductivity Calculation of Monolayer $Ti_3C_2T_x$

R=557 Ω

ρ=RS/L=557*3170*1.8/8000*10^{-9}=3.98*10^{-7} Ω·m

σ=1/R=1/2.86*10^{7}≈2.5 *10^{6} S/m

5. Calculation of EMI SE of the $Ti_3C_2T_x$ films.

(1) The reflection loss (SE_R) and absorption loss (SE_A) of conductive shielding can be calculated by the following equations.

$$SE_R(dB) = 20\log\frac{(\eta+\eta_0)^2}{4\eta\eta_0} \quad (1)$$

$$SE_A(dB) = 20\log e^{ad} = 20\left(\frac{d}{\delta}\right)\log e = 8.68\left(\frac{d}{\delta}\right) = 8.68d\sqrt{\pi f\mu\sigma} \quad (2)$$

$$\alpha = \omega\sqrt{\frac{\mu\varepsilon}{2}\left[\sqrt{1+(\frac{\sigma}{\omega\varepsilon})^2}-1\right]} \quad (3)$$

where η and η_0 are the impedances of the shield and air, respectively, σ and μ are the electrical conductivity and the magnetic permeability of the shield, respectively, f is the frequency of the incident electromagnetic waves, and ω is angular frequency and ε is dielectric permittivity.

(2) The EMI SE was calculated from the scattering parameters (S11 and S21) by the following formulas:

$$R = |S_{11}|^2 = |S_{22}|^2 \quad (4)$$

$$T = |S_{12}|^2 = |S_{21}|^2 \quad (5)$$

$$A + R + T = 1 \quad (6)$$

$$SE_T = SE_R + SE_A + SE_M \quad (7)$$

The total shielding effectiveness (SE_T) is known as the sum of contributions resulting from reflection (SE_R), absorption (SE_A) and multiple reflections (SE_M). In many cases, multi-reflection has been considered as an absorption because multiple internal reflections of electromagnetic waves are absorbed or dissipated as heat in the shielding materials. Therefore, the total SE (SE_T) can be rewritten as

$$SE_T = SE_R + SE_A \quad (8)$$

$$SE_T(dB) = -10\, log\, \frac{P_{out}}{P_{in}} = -10\, log\, T \quad (9)$$

$$SE_R(dB) = -10\log(1 - R) \quad (10)$$

$$SE_A(dB) = 10\log(\frac{1-R}{T}) \quad (11)$$

Where R, T and A are the reflection, transmission and absorption coefficients, respectively. P_{in} and P_{out} are the incident and transmitted power, respectively. SE_T, SE_R and SE_A are the total, reflective and absorptive EMI SE, respectively.

(3) Calculation of the Specific Shielding Effectiveness (*SSE/t*) of the $Ti_3C_2T_x$ films.

$$SSE/t = \frac{SE_T}{\rho t} \quad (12)$$

where "ρ" is the film density and "t" is the film thickness.

Supplementary Figures

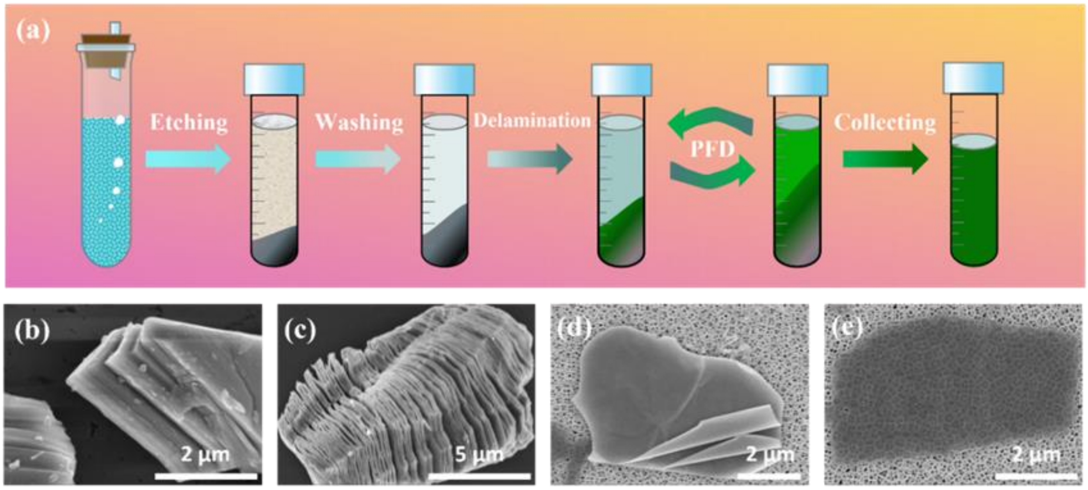

Supplementary Figure 1. (**a**) Schematic of $Ti_3C_2T_x$ preparation. SEM images of (**b**) Ti_3AlC_2, (**c**) multilayered structure, (**d**) few-layer MXene before delamination, and (**e**) monolayer MXene.

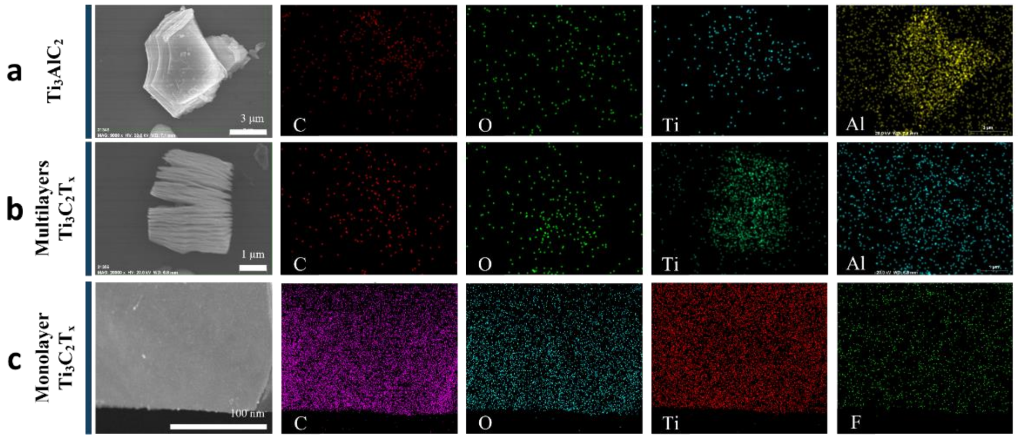

Supplementary Figure 2. SEM and elemental analysis images of (**a**) Ti_3AlC_2, (**b**) multilayer $Ti_3C_2T_x$, and (**c**) monolayer $Ti_3C_2T_x$.

The stacked arrangement of Ti_3C_2 sub-layers and Al atomic layers in the Ti_3AlC_2 MAX phase allows selective etching, starting from the etching of the initial Al atomic layer by LiF and HCl. As shown in Supplementary Fig. 1-2, acid etching allowed the successful removal of Al (Supplementary Table 1), and the $Ti_3C_2T_x$ MXene underwent interlayer expansion because of the intercalation of Li^+ ions. Because of the well-defined two-dimensional structure and flexibility of the $Ti_3C_2T_x$ MXene, the individual layers of the $Ti_3C_2T_x$ MXene stacks lift from the edges during the delamination process and can be delaminated by manual shaking. Therefore, when the multilayered MXene (tightly stacked layers) formed at the bottom of the sample tube are held in place, the energy of the water flow is concentrated on the $Ti_3C_2T_x$ MXenes at the surface. After the application of multiple PFD cycles, a high yield of large, exfoliated monolayers of the $Ti_3C_2T_x$ MXene can be obtained. A scanning electron microscopy (SEM) image of a single $Ti_3C_2T_x$ MXene layer prepared by the PFD method is shown in Supplementary Fig. 1e. The $Ti_3C_2T_x$ MXene is thin and transparent, and the porous alumina grid below the monolayer can be clearly seen.

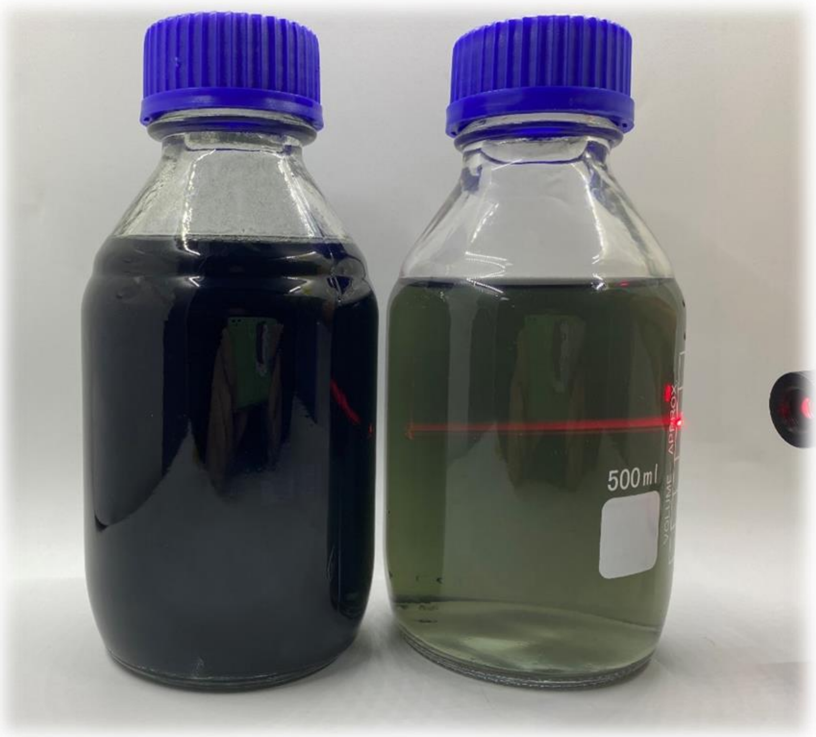

Supplementary Figure 3. Tyndall phenomenon in monolayer $Ti_3C_2T_x$ dispersions at low concentrations.

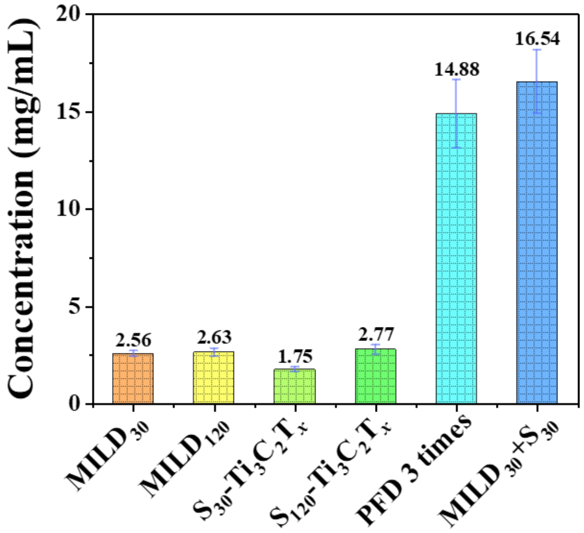

Supplementary Figure 4. Concentration of $Ti_3C_2T_x$ MXene dispersions obtained after PFD for different periods.

We evaluated the exfoliation efficiency of PFD using a vortex shaker to simulate the hand-shaking process. The concentration of the monolayer $Ti_3C_2T_x$ MXene solution obtained by the MILD method after 30 min of shaking was only 2.56 mg/mL, slightly higher than the literature-reported value, probably because mechanical shaking is more efficient than hand-shaking. However, with increase in the shaking time, there was no obvious improvement in exfoliation efficiency. This is because the impact of the vortex fluidic on the multilayer $Ti_3C_2T_x$ MXene is insufficient for delamination because both the monolayer $Ti_3C_2T_x$ MXene and fluid move together. Surprisingly, the delamination efficiency of ultrasonic treatment alone is about the same as that of the MILD method.

We also found that shaking combined with ultrasound-assisted delamination was also excellent. Further, the delamination yield can be increased by increasing the number of PFD cycles, reaching 61.2% after five cycles. In general, ultrasound, vortex shear caused by hand-shaking, and centrifugal processing can exfoliate multilayer MXenes. Furthermore, both sonication after sufficient shaking and redispersion after centrifugation (*i.e.*, *via* PFD) can substantially improve the delamination efficiency.

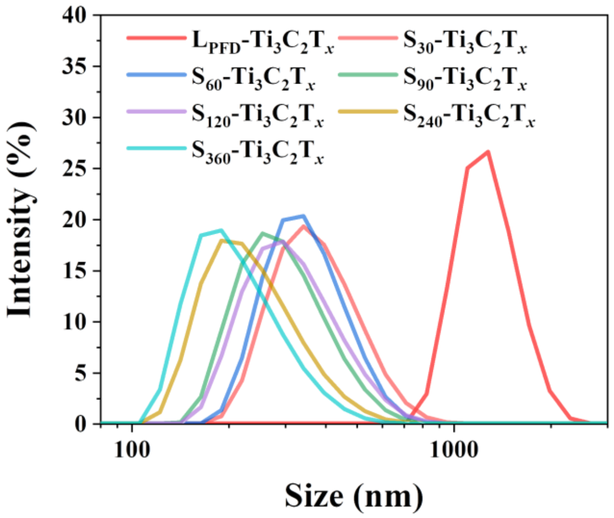

Supplementary Figure 5. Dynamic light scattering analysis of $Ti_3C_2T_x$ prepared by ultrasonic treatment for different periods.

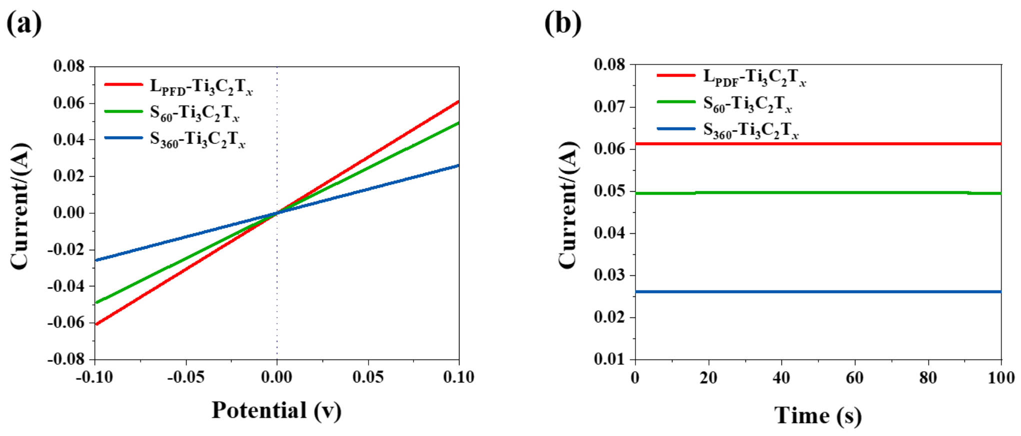

Supplementary Figure 6. Current curve corresponding to the voltage of $Ti_3C_2T_x$ MXene under (a) different voltage and (b) constant voltage conditions.

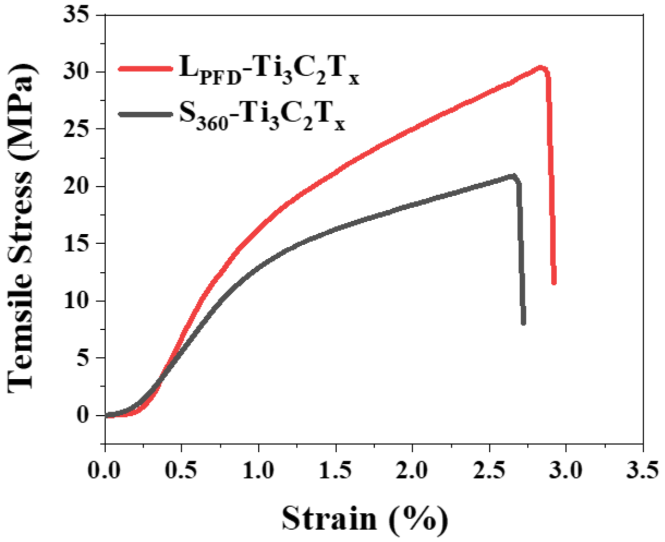

Supplementary Figure 7. Mechanical properties of thin films prepared from $Ti_3C_2T_x$ nanosheets of large and small sizes.

As reported previously, the application of ultrasound causes monolayer $Ti_3C_2T_x$ MXene nanosheets to break up, as well as the generation of defects. Therefore, in comparison to the ultrasound method, the PFD method is an efficient route to prepare large monolayers of $Ti_3C_2T_x$ MXenes with few of basal-plane defects.

Notice, sonication decreases the transverse dimension of $Ti_3C_2T_x$ MXene. However, the relationship between the ultrasound time and the transverse dimensions of MXene nanosheets has not been investigated to date. As shown in Supplementary Fig. 5 and Table S1, sonication causes the structural integrity of MXene nanosheets to be disrupted, resulting in a substantial reduction in the dimensions of the material. In particular, in the first 30 min of sonication, the size of the MXene nanosheets decrease rapidly. Along with the size reduction, the conductivity of the $Ti_3C_2T_x$ MXenes also decreases dramatically. Because electron transport inside the MXene nanosheet is almost barrier-free but the irregular accumulation of MXene nanosheets results in contact resistance between the nanosheets, the formation of smaller nanosheets leads to a decrease in conductivity Supplementary Fig. 6. In addition, the larger $Ti_3C_2T_x$ MXene sheets have greater tensile strength (Supplementary Fig. 7). Therefore, the PFD method provides an efficient way to prepare high-quality $Ti_3C_2T_x$ MXene sheets having high conductivity and high strength.

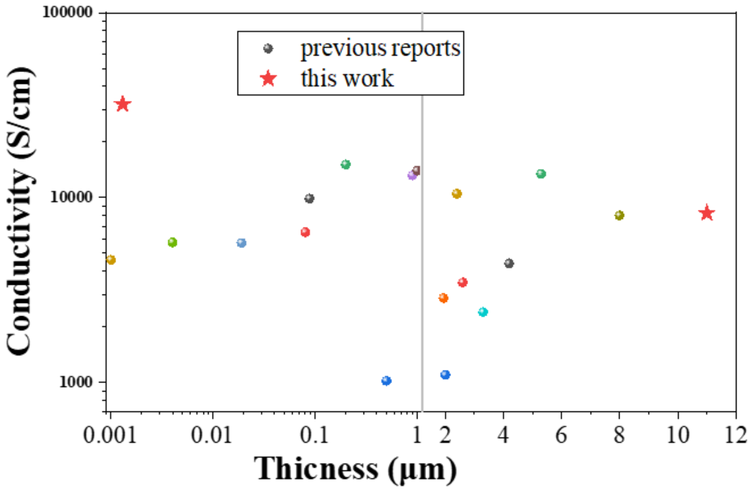

Supplementary Figure 8. Comprehensive comparison of the electrical conductivity of $Ti_3C_2T_x$ films prepared by PFD method with those reported in the literature with different thicknesses of $Ti_3C_2T_x$ films.

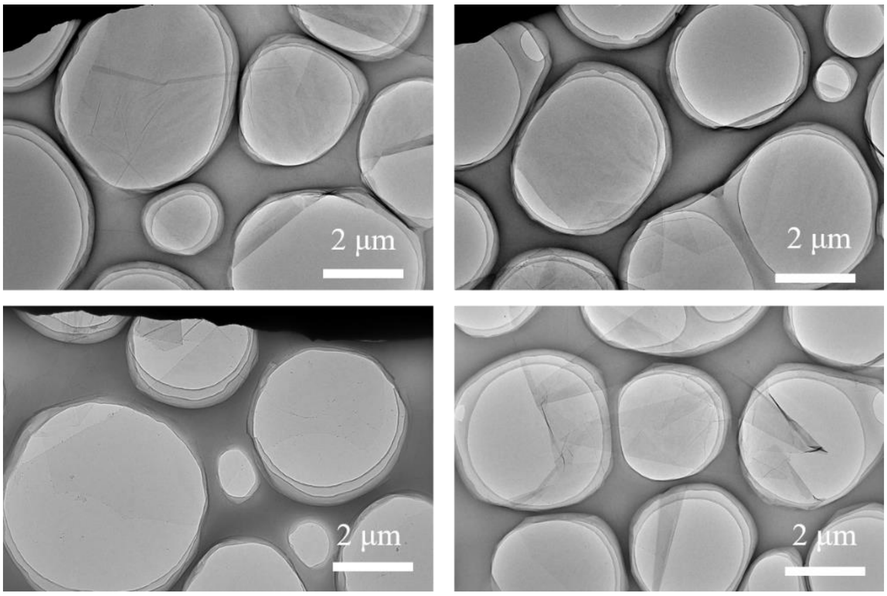

Supplementary Figure 9. TEM images of L_{PFD}-$Ti_3C_2T_x$.

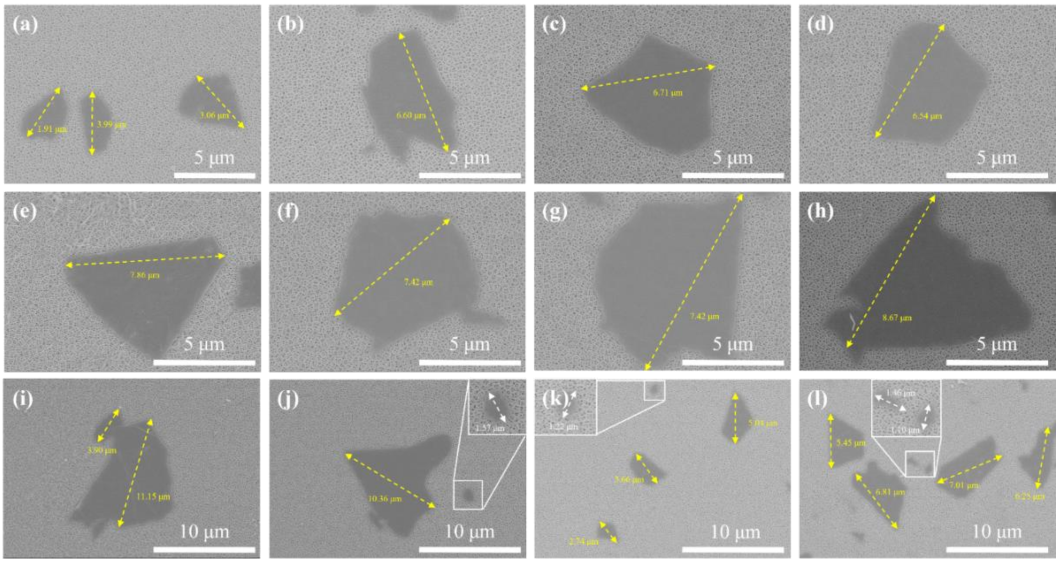

Supplementary Figure 10. Representative transverse dimensional statistics of SEM images of some monolayer $Ti_3C_2T_x$ nanosheets.

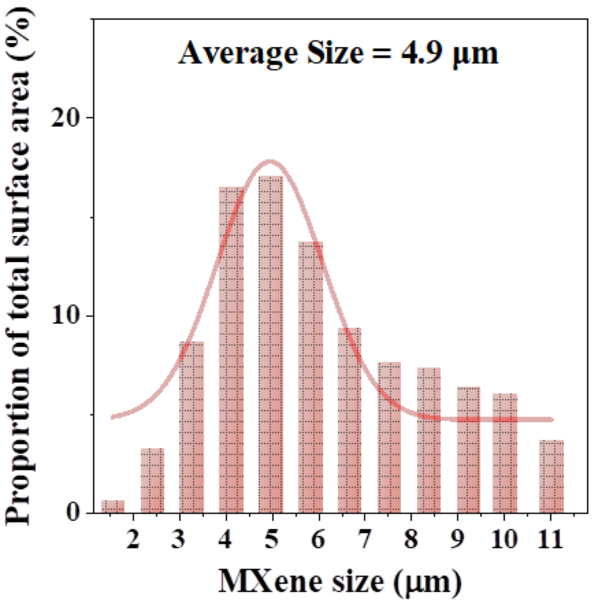

Supplementary Figure 11. Statistical plots of the lateral dimensions of 280 monolayer $Ti_3C_2T_x$ nanosheets and fitted curves.

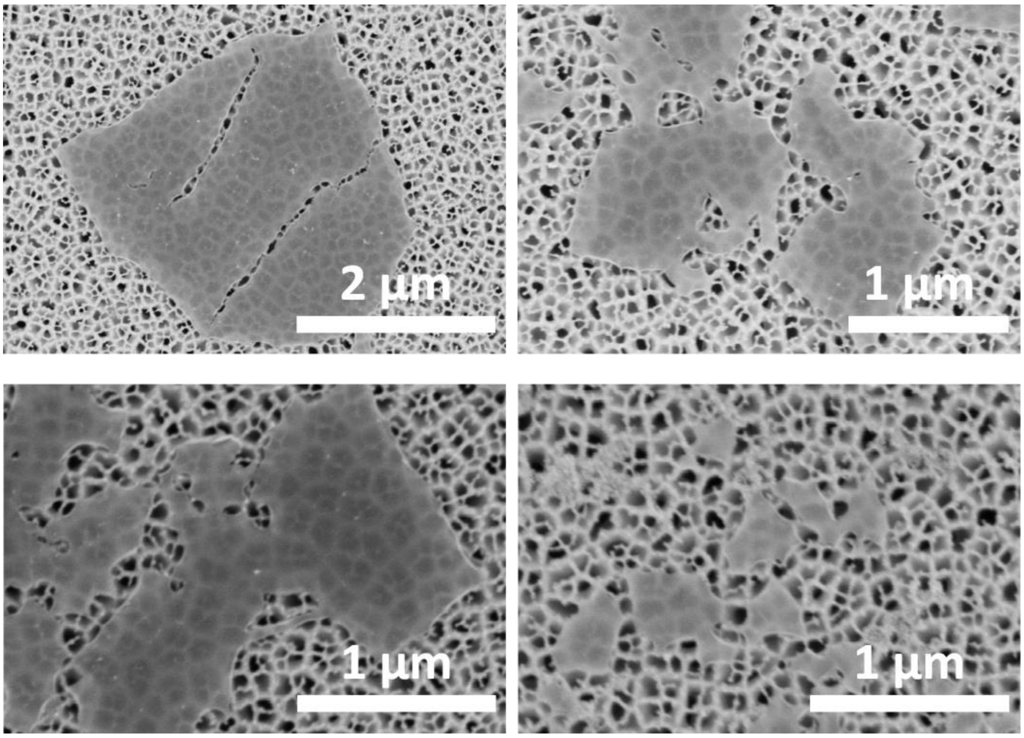

Supplementary Figure 12. SEM electron micrographs of broken monolayer $Ti_3C_2T_x$ nanosheets after ultrasound treatment.

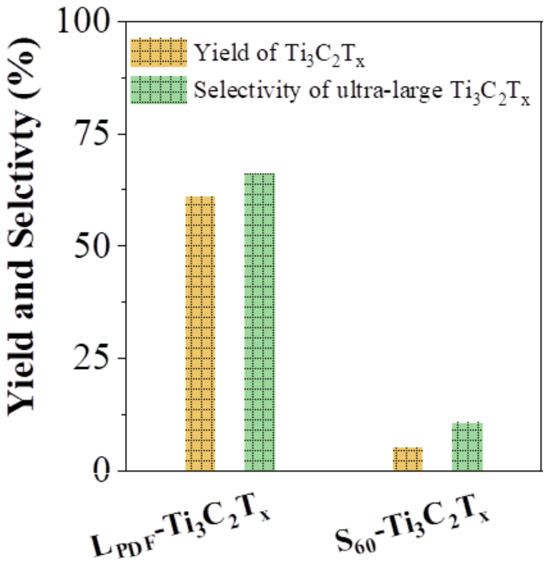

Supplementary Figure 13. Yields of the as-prepared L_{PFD}-$Ti_3C_2T_x$ MXene and S_{60}-$Ti_3C_2T_x$ MXene and the content of ultra-large $Ti_3C_2T_x$ nanosheets.

Here, ultra-large $Ti_3C_2T_x$ nanosheets were screened (as shown in the experimental section) by differential centrifugation and their solids content was calculated by freeze-drying. Among them, the ultra-large $Ti_3C_2T_x$ nanosheets solid content in L_{PFD}-$Ti_3C_2T_x$ MXene is 66.1% (wt/wt), and the ultra-large $Ti_3C_2T_x$ nanosheets content in S_{60}-$Ti_3C_2T_x$ MXene is only 10.8% (wt/wt).

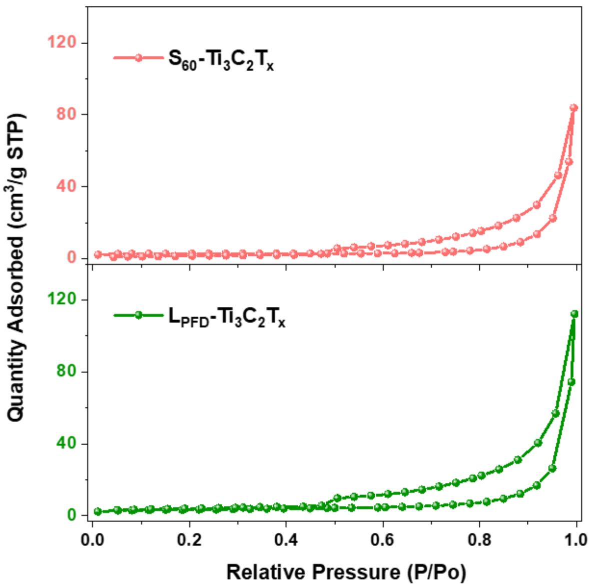

Supplementary Figure 14. N_2 adsorption–desorption isotherms of L_{PFD}-$Ti_3C_2T_x$ MXene and S_{60}-$Ti_3C_2T_x$ MXene.

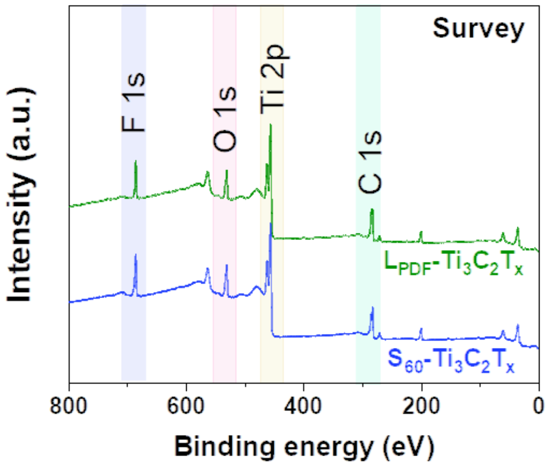

Supplementary Figure 15. XPS survey scan spectra of L_{PFD}-$Ti_3C_2T_x$ MXene and S_{60}-$Ti_3C_2T_x$ MXene.

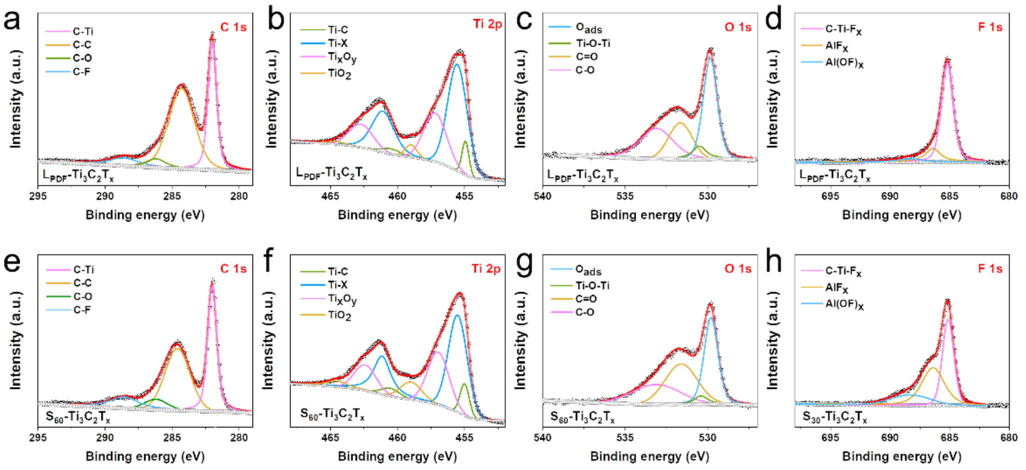

Supplementary Figure 16. High resolution (a) C 1s, (b) Ti 2p, (c) O 1s and (d) F 1s spectra of L_{PFD}-$Ti_3C_2T_x$ MXene and (e) C 1s, (f) Ti 2p, (g) O 1s and (h) F 1s spectra of S_{60}-$Ti_3C_2T_x$ MXene.

As seen from the high-resolution XPS spectra, the C 1s components centered at 282.0, 284.6, 286.2 and 288.6 eV can be assigned as C–Ti, C–C/C=C, C–O, and C–F bond, respectively[2]. The Ti 2p 3/2 spectrum consists of four kinds of titanium species. Four peaks centered at 454.9, 455.6, 457.2, and 459.0 eV arose from the Ti−C bond, Ti−X from substoichiometric TiC_x ($x < 1$), Ti ions in valence 3^+ or 2^+ (Ti_xO_y), and Ti ions with oxidized charge state (TiO_2), respectively[2-3]. The O 1s XPS spectrum consists of four peaks located at 533.1, 531.7, 530.5, and 529.8 eV, which correspond to the C−O, C=O, Ti−O−Ti bond, and the surface adsorbed O species, respectively[2, 4]. With respect to the high-resolution F 1s XPS spectra, three peaks at 685.2, 686.4 and 688.3 eV have been deconvoluted, which are ascribed to C−Ti−F_x species, Al−F_x, and $Al(OF)_x$ species, respectively[5].

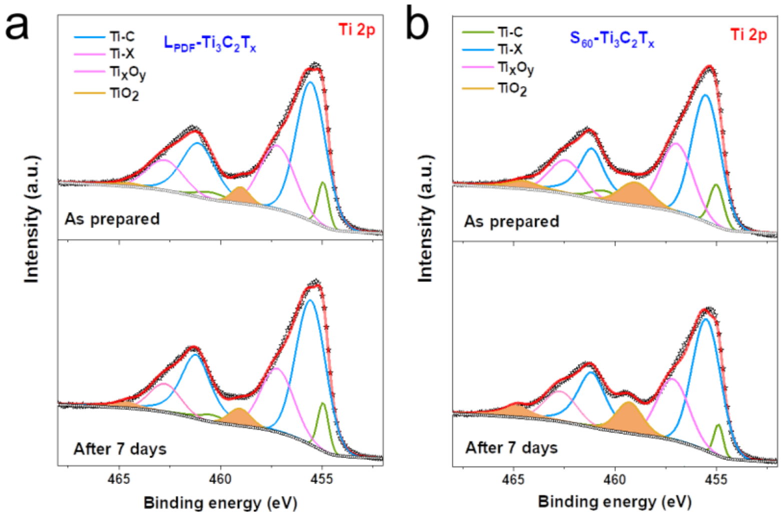

Supplementary Figure 17. High resolution of Ti 2p spectra of (a) L_{PFD}-$Ti_3C_2T_x$ MXene and (b) S_{60}-$Ti_3C_2T_x$ MXene. The MXene solution was stored at room temperature and under air condition.

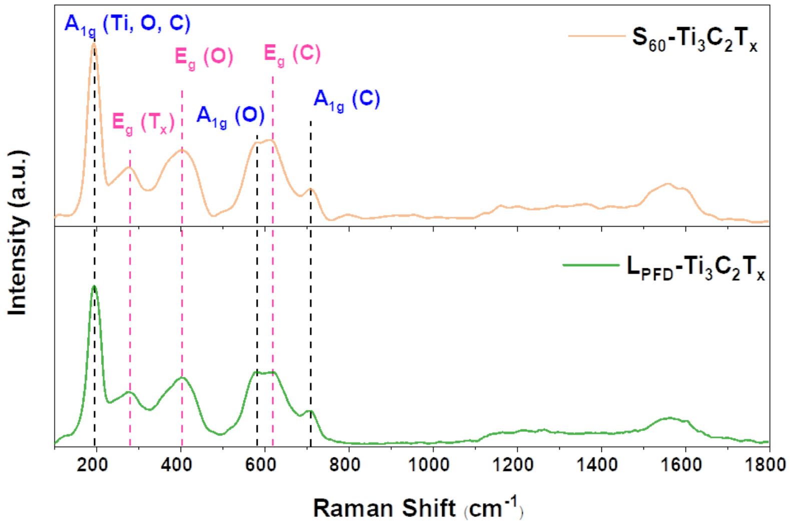

Supplementary Figure 18. Raman spectra of the as-prepared L_{PFD}-$Ti_3C_2T_x$ MXene and S_{60}-$Ti_3C_2T_x$ MXene.

The composition and purity of L_{PFD}-$Ti_3C_2T_x$ MXene and S_{60}-$Ti_3C_2T_x$ MXene can be also evaluated by Raman spectroscopy. The Raman peaks located at 195 cm^{-1}, 583 cm^{-1} and 704 cm^{-1} are assigned to the characteristic A1g symmetry out of plane vibration of (Ti, O, C), O atom and C atom, respectively[6]. The peaks at 275 cm^{-1}, 396 cm^{-1} and 616 cm^{-1} are attributed to the in-plane E_g vibration of H atom in the surface group (T_x), O atom and C atom, respectively[6].

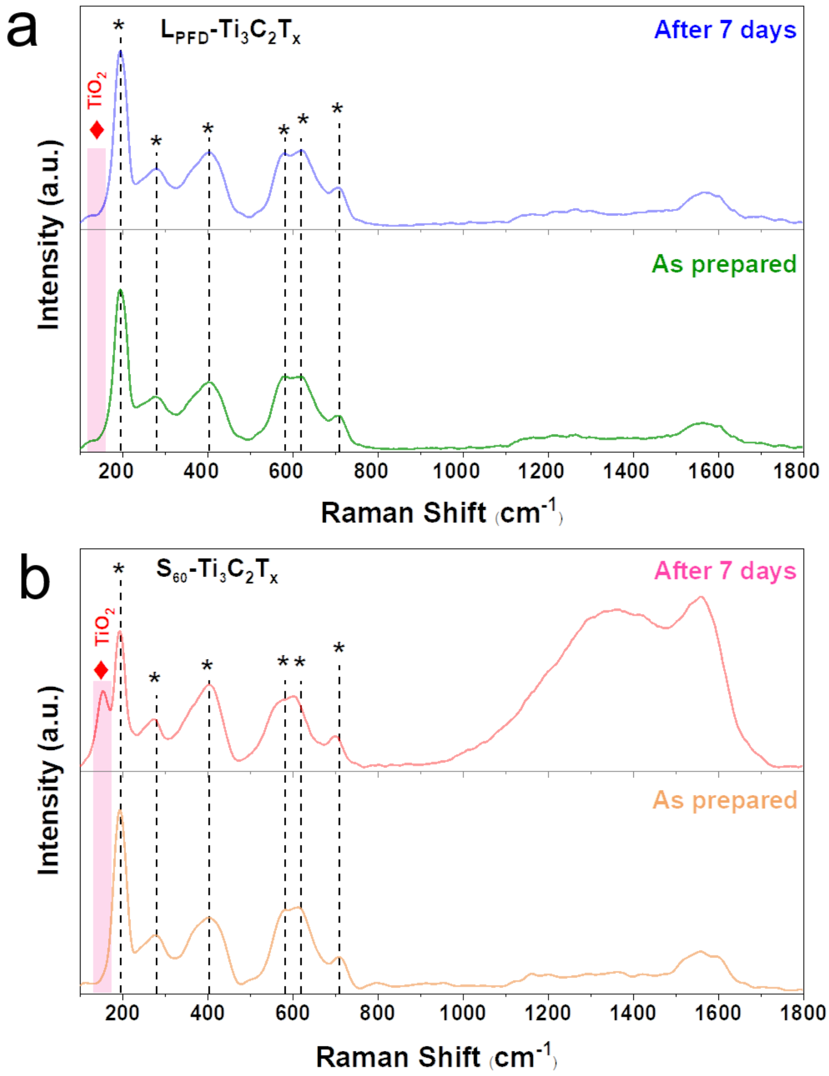

Supplementary Figure 19. Raman spectra of the (a) L_{PFD}-$Ti_3C_2T_x$ MXene and (b) S_{60}-$Ti_3C_2T_x$ MXene. The MXene solution was stored at room temperature and under air condition.

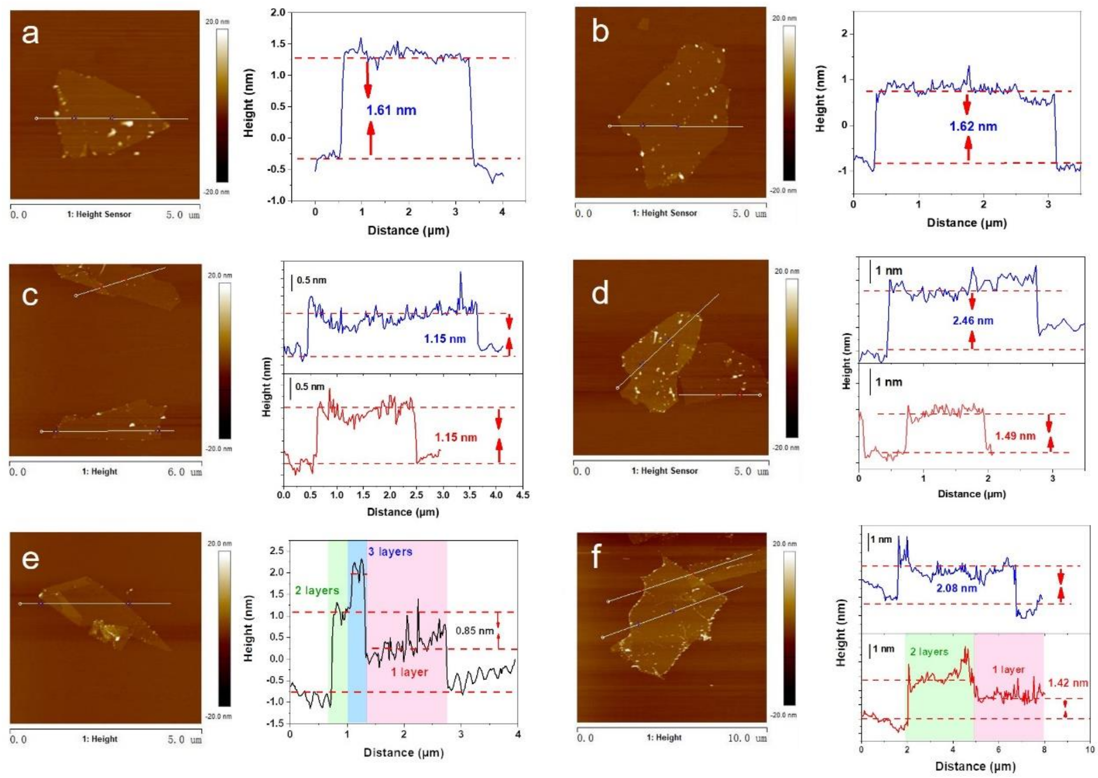

Supplementary Figure 20. Representative AFM images of L_{PFD}-$Ti_3C_2T_x$ MXene.

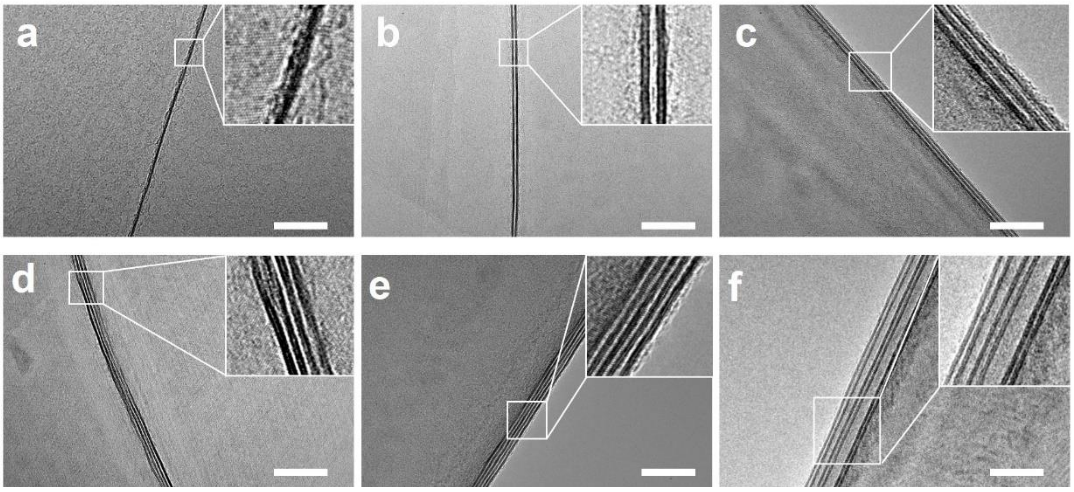

Supplementary Figure 21. Representative TEM images show 1-6 layers of flakes.

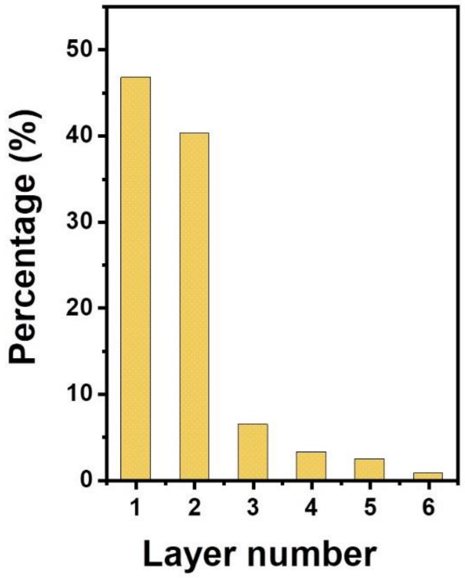

Supplementary Figure 22. Statistical analysis of layer number distribution of dispersed L_{PFD}-$Ti_3C_2T_x$ MXene through TEM measurements.

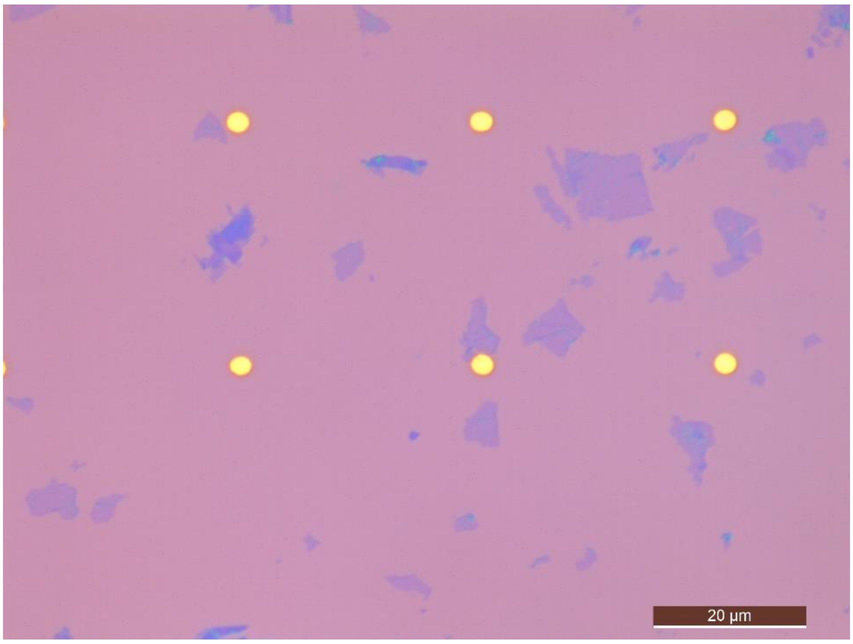

Supplementary Figure 23. Optical microscope image of L_{PFD}-$Ti_3C_2T_x$ MXene on SiO_2/Si substrate.

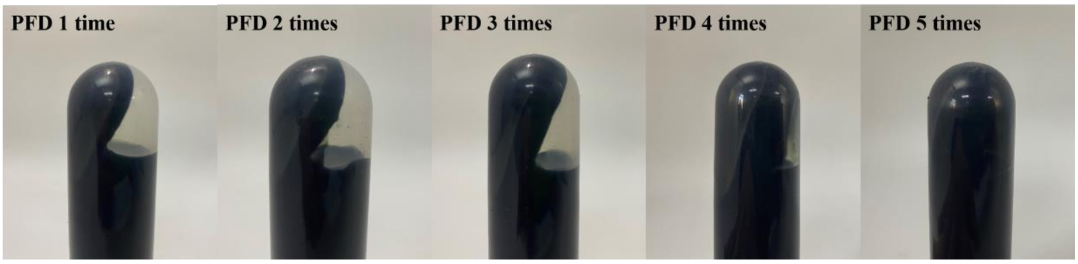

Supplementary Figure 24. Digital photographs of different PFD times.

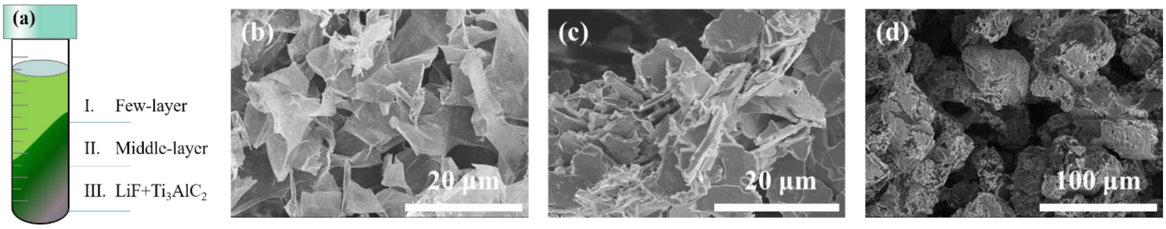

Supplementary Figure 25. (**a**) Schematic of the solid and supernatant after centrifugation, and SEM images of (**b**) few-layer, (**c**) meddle-layer, and (**d**) LiF+Ti$_3$AlC$_2$.

With increase in the number of PFD cycles, the precipitate became gradually and obviously divided into several parts. The presence of different components in the precipitate is also indicated by the differences in color. Therefore, we freeze-dried and collected the different portions of the precipitate for further analysis. Importantly, the different components are separated spontaneously during the centrifugation process, and impurities and raw materials were not detected in the XRD patterns of the upper layer and the final product.

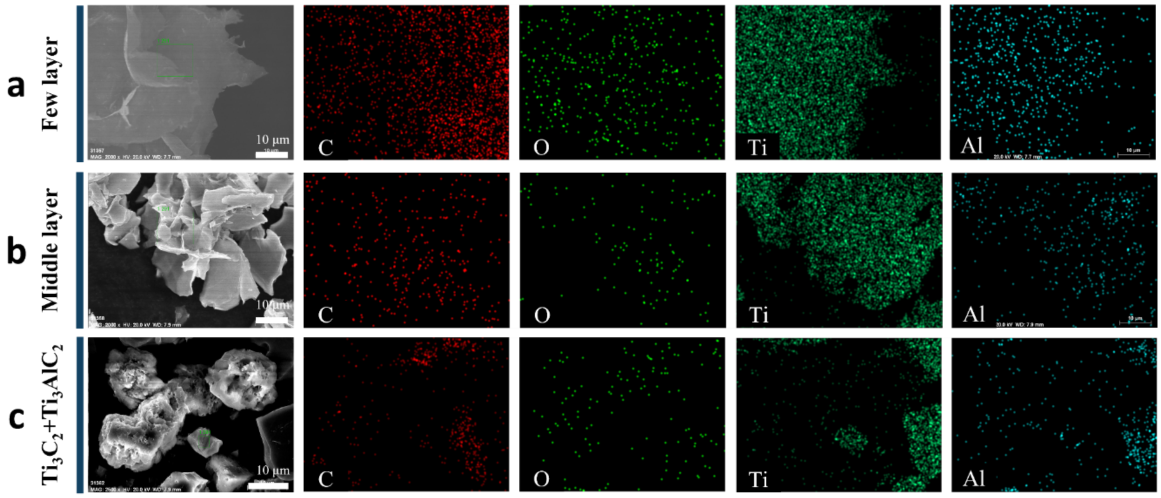

Supplementary Figure 26. SEM and elemental analysis images of different locations of centrifugal precipitation. (**a**) few-layer, (**b**) middle-layer, and (**c**) LiF+Ti_3AlC_2.

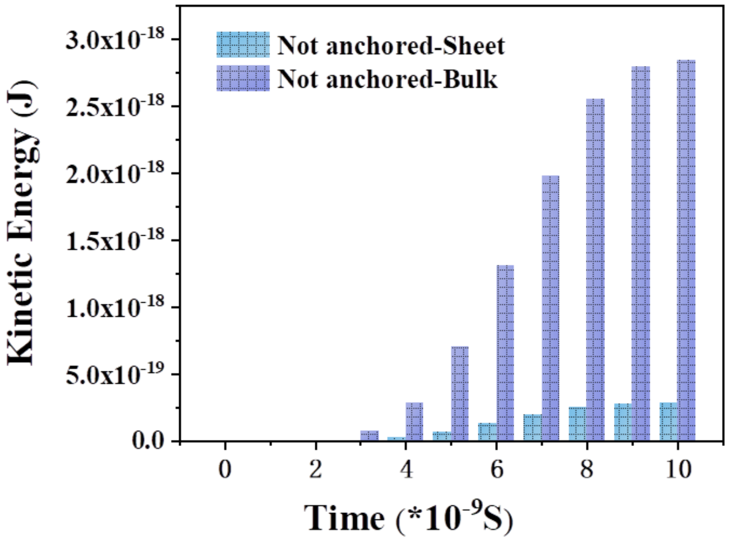

Supplementary Figure 27. Kinetic energy acquired by the surface layer and the block during delamination *via* the MILD method.

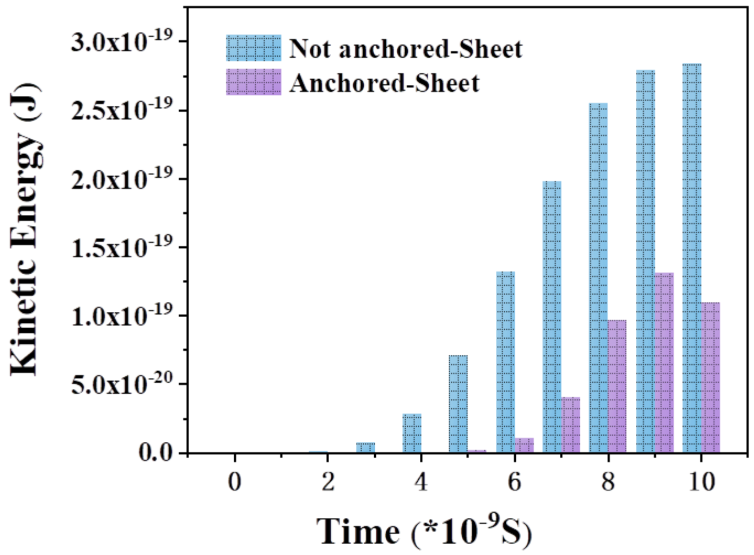

Supplementary Figure 28. Kinetic energy acquired by the surface layer during delamination *via* MILD and PFD methods.

Supplementary Table 1. Z-Average dimensions of $Ti_3C_2T_x$ for different sonication times.

Entry	Ultrasound time (min)	Z-Average (d.nm)
1	0	3768
2	30	419.4
3	60	299.4
4	90	233.8
5	120	209.4
6	150	192.7
7	180	189.4
8	210	158.6
9	240	150.3
10	270	140.2
11	300	137.8
12	330	133.2
13	360	130.1
14	420	127.9
15	480	113.8

All data were obtained using dynamic light scattering tests.

Supplementary Table 2. Conductivity of various $Ti_3C_2T_x$ MXene materials in the literature [1a, 7].

Materials	Conductivity (S/cm)	Thickness (μm)	Average flake size (μm)	Fabrication method	Ref.
Graphite-$Ti_3C_2T_x$	4400 ± 110	4.2	/	/	[1a]
TiC-$Ti_3C_2T_x$	3480 ± 60	2.6	/	/	
lampblack-$Ti_3C_2T_x$	1020 ± 50	0.5	/	/	
$Ti_3C_2T_x$	15,100 ± 160	0.2	≈10	after drying at 200 °C	[7a]
$Ti_3C_2T_x$	13,200 ± 130	0.9	≈10	after drying at 200 °C	
$Ti_3C_2T_x$	10,500 ± 240	2.4	≈10	after drying at 200 °C	
$Ti_3C_2T_x$	4,566	/	/	/	[7b]
$Ti_3C_2T_x$	2402.4 ± 3.5	3.3	/	/	[7c]
L-$Ti_3C_2T_x$	9,490	/	1	filtered	[7d]
S-$Ti_3C_2T_x$	4080	/	0.34	filtered	
$Ti_3C_2T_x$	1,250	1 to 21	/	vacuum drying	[7e]
$Ti_3C_2T_x$	14,000	1	/	/	[7f]
Ti_3C_2	8000	8	/	filtered freestanding film	[7h]
Ti_3C_2	2,863	1.94	≈1	/	[7g]
Ti_3C_2	5,682	0.019		etched with NH_4HF_2	[7i]
$Ti_3C_2T_x$ films	5736	0.0004	3.2	vacuum annealing at 200 °C	[7j]
$Ti_3C_2T_x$ films	9880	0.0088	3.2	vacuum annealing at 200 °C	
$Ti_3C_2T_x$	6500	0.08		Spin casting	[7k]
L-$Ti_3C_2T_x$	2508	/	3-6	Spray-coating	[7l]
s-$Ti_3C_2T_x$	778	/	1	Spray-coating	
$Ti_3C_2T_x$	230	/	3-4	few-layer	[7m]
$Ti_3C_2T_z$	1100	2	up to 1	/	[7o]
PS-1	1000	/	0.13±0.06	/	[7n]
BS-0	5000	/	4.4±1.5	/	
$Ti_3C_2T_x$	4000	/	3-5	microwave synthesis	[7p]
$Ti_3C_2T_z$	5857±680	/	6	/	[7q]
$Ti_3C_2T_x$	123	/	/	/	[7s]
L-$Ti_3C_2T_x$ fibers	7750	/	3.1	/	[7r]
S-$Ti_3C_2T_x$ fibers	3512	/	0.31	/	
L-$Ti_3C_2T_x$	13440	5.3	4	/	[7u]
$Ti_3C_2T_x$	24000		1.2	/	[7t]
$Ti_3C_2T_x$	4600 ± 1100	0.001	4-15		[7v]
L_{PFD}-$Ti_3C_2T_x$	8260	11	4.9	/	This work
L_{PFD}-$Ti_3C_2T_x$	25000	0.0018	4.9	/	

Supplementary Table 3. Comprehensive parameters for the preparation of $Ti_3C_2T_x$ MXene materials by various strategies in the literature [7f, 7m, 7o, 7t, 8].

Materials	Yield (%)	MXene size (μm)	Conductivity (S/cm)	Fabrication method	Ref.
$Ti_3C_2T_x$	12	up to 1	1100	Acoustic synthesis	[7o]
	39	55	9771	freezingand-thawing approach	[8j]
	60	2.4	1330	Delaminated in a binary aqueous electrolyte	[8a]
	74	0.3-1	405	Hydrothermal assisted intercalation	[8c]
	85	3-4	2300	Exfoliated by $NaHF_2$	[7m]
	80	1.5	36.7	Etched with HCl and LiF	[8d]
	45-50	0.35	200	Etched from Ti_3SiC_2	[8e]
	52	2.5	6220	Scalable synthesis	[8f]
	60	1.8	24000	EN-MILD	[7t]
	71	1.8	1250	Iodine etching	[8g]
	40.2	3.02	8672	Organic solvent-assisted intercalation and collection	[8h]
	65	0.36	3174	Decreasing precursor size	[8i]
	61.2	4.9	25000	Power-focused delamination method	This work

Supplementary Table 4: The element content corresponds to each part of Supplementary Figure 26.

	**C**	**O**	**Ti**	**Al**
**Few layer**	32.01	19.02	47.43	1.54
**Middle layer**	15.05	8.06	74.2	2.68
**Ti_3C_2+Ti_3AlC_2**	13.50	11.56	69.62	5.33

Supplementary Table 5. Comprehensive performance of various shielding materials[8b, 9].

Filler	Matrix	Content (wt%)	Thickness (cm)	EMI SE (dB)	SSE/t (dB*cm^2*g^{-1})	Ref.
Graphene	PS	7	0.25	45.1	692	[9a]
	PEI	10	0.23	12.8	191.3	[9b]
	PS	30	0.2	29	257.6	[9c]
	PI	16	0.08	21	11712	[9d]
		Bulk	0.03	25.2	14000	[9e]
	PDMS	0.8	0.1	19.98	3330	[9f]
	Polymer	25	0.005	21.5	10652	[9g]
Graphene-iron pentacarbonyl film	/	/	0.03	38	10555	[9h]
Graphene film	/	/	0.0031	130	25727	[9i]
Graphene-CNT	/	/	0.36	66	31000	[9j]
CNT	Cellulose	40	0.015	35	1372.4	[9k]
	ANF	27	0.0168	22.7	33528.3	[9l]
	TOCNFs	75	0.015	46.4	4017.3	[9m]
	natural rubber	6.4	0.13	37.33	308.1	[9n]
CNT-sponge	/	Bulk	0.24	22	4622	[9o]
MWCNT	WPU	76.2	0.1	21.1	5410	[9p]
CuNi	/	/	0.15	25	690	[9q]
CuNi-CNT	/	/	0.15	54.6	1580	[9q]
Cu foil	/	Bulk	0.001	70	7812	[9r]
Al foil	/	Bulk	0.0008	66	30555	[9r]
rGO	cellulose fiber	33	0.5	36.4	24761	[9s]
rGO-Fe_3O_4@SiO_2	/	/	0.027	32	12608.4	[9t]
MXene	ANF	40	0.0011	21	9555.7	[9u]
	Montmorillonite	90	0.0025	67	10156.3	[9v]
	CNF	50	0.0035	39.6	7029	[9w]
	CNF	90	0.0047	24	2647	[9x]
	ANF	90.91	0.0012	34.71	21971.37	[9y]
	polyurethane	30	0.00093	28.5	18756.17	[9z]
	TOCNFs	50	0.0047	32.7	4761	[9aa]
	ANF	40	0.0022	19.43	7434.1	[8b]
	ANF	40	0.0009	40.7	28190	[9ab]
$Ti_3C_2T_x$	/	Bulk	0.0011	68	25863	[9r]
	SA	90	0.0008	57	30830	[9r]
MXene	/	/	0.001	54.42	35418.7	This Work
	/	/	0.001	50.11	30513.3	
	/	/	0.001	39.77	26426.1	

[1] a) C. E. Shuck, M. Han, K. Maleski, K. Hantanasirisakul, S. J. Kim, J. Choi, W. E. B. Reil, Y. Gogotsi, *ACS Appl. Nano Mater.* **2019**, *2* (6), 3368, https://doi.org/10.1021/acsanm.9b00286; b) M. Alhabeb, K. Maleski, B. Anasori, P. Lelyukh, L. Clark, S. Sin, Y. Gogotsi, *Chem. Mater.* **2017**, *29* (18), 7633, https://doi.org/10.1021/acs.chemmater.7b02847.

[2] C. Peng, X. Yang, Y. Li, H. Yu, H. Wang, F. Peng, *ACS Appl. Mater. Interfaces* **2016**, *8* (9), 6051, https://doi.org/10.1021/acsami.5b11973.

[3] R. B. Rakhi, B. Ahmed, M. N. Hedhili, D. H. Anjum, H. N. Alshareef, *Chem. Mater.* **2015**, *27* (15), 5314, https://doi.org/10.1021/acs.chemmater.5b01623.

[4] J. H. Woo, N. H. Kim, S. I. Kim, O.-K. Park, J. H. Lee, *Compos. B Eng.* **2020**, *199*, 108205, https://doi.org/10.1016/j.compositesb.2020.108205.

[5] J. Halim, K. M. Cook, M. Naguib, P. Eklund, Y. Gogotsi, J. Rosen, M. W. Barsoum, *Appl. Surf. Sci.* **2016**, *362*, 406, https://doi.org/10.1016/j.apsusc.2015.11.089.

[6] a) O. B. Seo, S. Saha, N. H. Kim, J. H. Lee, *J. Membr. Sci.* **2021**, *640*, 119839, https://doi.org/10.1016/j.memsci.2021.119839; b) A. Sarycheva, Y. Gogotsi, *Chem. Mater.* **2020**, *32* (8), 3480, https://doi.org/10.1021/acs.chemmater.0c00359.

[7] a) J. Zhang, N. Kong, S. Uzun, A. Levitt, S. Seyedin, P. A. Lynch, S. Qin, M. Han, W. Yang, J. Liu, X. Wang, Y. Gogotsi, J. M. Razal, *Adv. Mater.* **2020**, *32* (23), 2001093, https://doi.org/10.1002/adma.202001093; b) J. Yan, C. E. Ren, K. Maleski, C. B. Hatter, B. Anasori, P. Urbankowski, A. Sarycheva, Y. Gogotsi, *Adv. Funct. Mater.* **2017**, *27* (30), 1701264, https://doi.org/10.1002/adfm.201701264; c) Z. Ling, C. E. Ren, M.-Q. Zhao, J. M.

Giammarco, M. W. Barsoum, Y. Gogotsi, J. Yang, J. Qiu, *PNAS* **2014**, *111* (47), 16676, https://doi.org/doi.org/10.1073/pnas.1414215111; d) S. Uzun, S. Seyedin, A. L. Stoltzfus, A. S. Levitt, M. Alhabeb, M. Anayee, C. J. Strobel, J. M. Razal, G. Dion, Y. Gogotsi, *Adv. Funct. Mater.* **2019**, *29* (45), 1905015, https://doi.org/10.1002/adfm.201905015; e) H. Huang, H. Su, H. Zhang, L. Xu, X. Chu, C. Hu, H. Liu, N. Chen, F. Liu, W. Deng, B. Gu, H. Zhang, W. Yang, *Adv. Electron. Mater.* **2018**, *4* (8), 1800179, https://doi.org/10.1002/aelm.201800179; f) S. A. Mirkhani, A. Shayesteh Zeraati, E. Aliabadian, M. Naguib, U. Sundararaj, *ACS Appl. Mater. Interfaces* **2019**, *11* (20), 18599, https://doi.org/10.1021/acsami.9b00393; g) Z. Ma, X. Zhou, W. Deng, D. Lei, Z. Liu, *ACS Appl. Mater. Interfaces* **2018**, *10* (4), 3634, https://doi.org/10.1021/acsami.7b17386; h) A. Sarycheva, A. Polemi, Y. Liu, K. Dandekar, B. Anasori, Y. Gogotsi, *Sci. Adv.* **2018**, *4*, e0920, https://doi.org/10.1126/sciadv.aau0920; i) J. Halim, M. R. Lukatskaya, K. M. Cook, J. Lu, C. R. Smith, L. A. Naslund, S. J. May, L. Hultman, Y. Gogotsi, P. Eklund, M. W. Barsoum, *Chem. Mater.* **2014**, *26* (7), 2374, https://doi.org/10.1021/cm500641a; j) C. Zhang, B. Anasori, A. Seral-Ascaso, S.-H. Park, N. McEvoy, A. Shmeliov, G. S. Duesberg, J. N. Coleman, Y. Gogotsi, V. Nicolosi, *Adv. Mater.* **2017**, *29* (36), 1702678, https://doi.org/10.1002/adma.201702678; k) A. D. Dillon, M. J. Ghidiu, A. L. Krick, J. Griggs, S. J. May, Y. Gogotsi, M. W. Barsoum, A. T. Fafarman, *Adv. Funct. Mater.* **2016**, *26* (23), 4162, https://doi.org/10.1002/adfm.201600357; l) Y.-Y. Peng, B. Akuzum, N. Kurra, M.-Q. Zhao, M. Alhabeb, B. Anasori, E. C. Kumbur, H. N. Alshareef, M.-D. Ger, Y. Gogotsi, *Energy Environ. Sci.* **2016**, *9* (9), 2847, https://doi.org/10.1039/C6EE01717G; m) Y. Zhao, M. Zhang, H. Yan, Y. Feng, X. Zhang, R. Guo, *J. Mater. Chem. A* **2021**, *9* (15), 9593, https://doi.org/10.1039/d0ta12231a; n) K. Maleski, C. E. Ren, M. Q. Zhao, B. Anasori, Y.

Gogotsi, *ACS Appl. Mater. Interfaces* **2018**, *10* (29), 24491, https://doi.org/10.1021/acsami.8b04662; o) A. E. Ghazaly, H. Ahmed, A. R. Rezk, J. Halim, P. O. A. Persson, L. Y. Yeo, J. Rosen, *ACS Nano* **2021**, *15* (3), 4287, https://doi.org/10.1021/acsnano.0c07242; p) J. Zhu, J. Zhang, R. Lin, B. Fu, C. Song, W. Shang, P. Tao, T. Deng, *Chem. Commun.* **2021**, *57* (94), 12611, https://doi.org/10.1039/d1cc04989e; q) S. Jolly, M. P. Paranthaman, M. Naguib, *Mater. Today Adv.* **2021**, *10*, 100139, https://doi.org/10.1016/j.mtadv.2021.100139; r) J. Zhang, S. Uzun, S. Seyedin, P. A. Lynch, B. Akuzum, Z. Wang, S. Qin, M. Alhabeb, C. E. Shuck, W. Lei, E. C. Kumbur, W. Yang, X. Wang, G. Dion, J. M. Razal, Y. Gogotsi, *ACS Cent. Sci.* **2020**, *6* (2), 254, https://doi.org/10.1021/acscentsci.9b01217; s) M.-Q. Zhao, C. E. Ren, Z. Ling, M. R. Lukatskaya, C. Zhang, K. L. Van Aken, M. W. Barsoum, Y. Gogotsi, *Adv. Mater.* **2015**, *27* (2), 339, https://doi.org/10.1002/adma.201404140; t) A. Shayesteh Zeraati, S. A. Mirkhani, P. Sun, M. Naguib, P. V. Braun, U. Sundararaj, *Nanoscale* **2021**, *13*, 3572, https://doi.org/10.1039/d0nr06671k; u) S. Uzun, M. K. Han, C. J. Strobel, K. Hantanasirisakul, A. Goad, G. Dion, Y. Gogotsi, *Carbon* **2021**, *174*, 382, https://doi.org/10.1016/j.carbon.2020.12.021; v) A. Lipatov, M. Alhabeb, M. R. Lukatskaya, A. Boson, Y. Gogotsi, A. Sinitskii, *Adv. Electron. Mater.* **2016**, *2* (12), e1600255, https://doi.org/10.1002/aelm.201600255.

[8] a) S. Yang, P. Zhang, F. Wang, A. G. Ricciardulli, M. R. Lohe, P. W. M. Blom, X. Feng, *Angew. Chem., Int. Ed.* **2018**, *57* (47), 15491, https://doi.org/10.1002/anie.201809662; b) F. Xie, F. F. Jia, L. H. Zhuo, Z. Q. Lu, L. M. Si, J. Z. Huang, M. Y. Zhang, Q. Ma, *Nanoscale* **2019**, *11* (48), 23382, https://doi.org/10.1039/c9nr07331k; c) F. Han, S. Luo, L. Xie, J. Zhu, W. Wei,

X. Chen, F. Liu, W. Chen, J. Zhao, L. Dong, K. Yu, X. Zeng, F. Rao, L. Wang, Y. Huang, *ACS Appl. Mater. Interfaces* **2019**, *11* (8), 8443, https://doi.org/10.1021/acsami.8b22339; d) K. Rajavel, X. Yu, P. Zhu, Y. Hu, R. Sun, C. Wong, *ACS Appl. Mater. Interfaces* **2020**, *12* (44), 49737, https://doi.org/10.1021/acsami.0c12835; e) M. Alhabeb, K. Maleski, T. S. Mathis, A. Sarycheva, C. B. Hatter, S. Uzun, A. Levitt, Y. Gogotsi, *Angew. Chem., Int. Ed.* **2018**, *57* (19), 5444, https://doi.org/10.1002/anie.201802232; f) C. E. Shuck, A. Sarycheva, M. Anayee, A. Levitt, Y. Zhu, S. Uzun, V. Balitskiy, V. Zahorodna, O. Gogotsi, Y. Gogotsi, *Adv. Eng. Mater.* **2020**, *22* (3), e1901241, https://doi.org/10.1002/adem.201901241; g) H. H. Shi, P. P. Zhang, Z. C. Liu, S. Park, M. R. Lohe, Y. P. Wu, A. S. Nia, S. Yang, X. L. Feng, *Angew. Chem., Int. Ed.* **2021**, *60* (16), 8689, https://doi.org/10.1002/anie.202015627; h) D. Qu, Y. Jian, L. Guo, C. Su, N. Tang, X. Zhang, W. Hu, Z. Wang, Z. Zhao, P. Zhong, P. Li, T. Du, H. Haick, W. Wu, *Nano-Micro Lett.* **2021**, *13* (1), 188, https://doi.org/10.1007/s40820-021-00705-4; i) J. Xu, J. Zhu, C. Gong, Z. Guan, D. Yang, Z. Shen, W. Yao, H. Wu, *Chin. Chem. Lett.* **2020**, *31* (4), 1039, https://doi.org/10.1016/j.cclet.2020.02.050; j) X. Huang, P. Wu, *Adv. Funct. Mater.* **2020**, *30* (12), 1910048, https://doi.org/10.1002/adfm.201910048.

[9] a) D.-X. Yan, H. Pang, B. Li, R. Vajtai, L. Xu, P.-G. Ren, J.-H. Wang, Z.-M. Li, *Adv. Funct. Mater.* **2015**, *25* (4), 559, https://doi.org/10.1002/adfm.201403809; b) J. Ling, W. Zhai, W. Feng, B. Shen, J. Zhang, W. Zheng, *ACS Appl. Mater. Interfaces* **2013**, *5* (7), 2677, https://doi.org/10.1021/am303289m; c) D.-X. Yan, P.-G. Ren, H. Pang, Q. Fu, M.-B. Yang, Z.-M. Li, *J. Mater. Chem.* **2012**, *22* (36), 18772, https://doi.org/10.1039/c2jm32692b; d) Y. Li, X. L. Pei, B. Shen, W. T. Zhai, L. H. Zhang, W. G. Zheng, *RSC Adv.* **2015**, *5* (31), 24342, https://doi.org/10.1039/c4ra16421k; e) B. Shen, Y. Li, D. Yi, W. T. Zhai, X. C. Wei, W. G.

Zheng, *Carbon* **2016**, *102*, 154, https://doi.org/10.1016/j.carbon.2016.02.040; f) Z. P. Chen, C. Xu, C. Q. Ma, W. C. Ren, H. M. Cheng, *Adv. Mater.* **2013**, *25* (9), 1296, https://doi.org/10.1002/adma.201204196; g) L. F. Wei, W. B. Zhang, J. Z. Ma, S. L. Bai, Y. J. Ren, C. Liu, D. Simion, J. B. Qin, *Carbon* **2019**, *149*, 679, https://doi.org/10.1016/j.carbon.2019.04.058; h) J. Liu, H. B. Zhang, Y. F. Liu, Q. W. Wang, Z. S. Liu, Y. W. Mai, Z. Z. Yu, *Compos. Sci. Technol.* **2017**, *151*, 71, https://doi.org/10.1016/j.compscitech.2017.08.005; i) E. Z. Zhou, J. B. Xi, Y. J. Liu, Z. Xu, Y. Guo, L. Peng, W. W. Gao, J. Ying, Z. C. Chen, C. Gao, *Nanoscale* **2017**, *9* (47), 18613, https://doi.org/10.1039/c7nr07030f; j) Q. Song, F. Ye, X. W. Yin, W. Li, H. J. Li, Y. S. Liu, K. Z. Li, K. Y. Xie, X. H. Li, Q. G. Fu, L. F. Cheng, L. T. Zhang, B. Q. Wei, *Adv. Mater.* **2017**, *29* (31), 1701583, https://doi.org/10.1002/adma.201701583; k) L. Q. Zhang, B. Yang, J. Teng, J. Lei, D. X. Yan, G. J. Zhong, Z. M. Li, *J. Mater. Chem. C* **2017**, *5* (12), 3130, https://doi.org/10.1039/c6tc05516h; l) P. Y. Hu, J. Lyu, C. Fu, W. B. Gong, J. H. Liao, W. B. Lu, Y. P. Chen, X. T. Zhang, *ACS Nano* **2020**, *14* (1), 688, https://doi.org/10.1021/acsnano.9b07459; m) H. R. Zhang, X. W. Sun, Z. G. Heng, Y. Chen, H. W. Zou, M. Liang, *Ind. Eng. Chem. Res.* **2018**, *57* (50), 17152, https://doi.org/10.1021/acs.iecr.8b04573; n) Y. H. Zhan, M. Oliviero, J. Wang, A. Sorrentino, G. G. Buonocore, L. Sorrentino, M. Lavorgna, H. S. Xia, S. Iannace, *Nanoscale* **2019**, *11* (3), 1011, https://doi.org/10.1039/c8nr07351a; o) M. Crespo, M. Gonzalez, A. L. Elias, L. P. Rajukumar, J. Baselga, M. Terrones, J. Pozuelo, *Phys. Status Solidi RRL* **2014**, *8* (8), 698, https://doi.org/10.1002/pssr.201409151; p) Z. H. Zeng, H. Jin, M. J. Chen, W. W. Li, L. C. Zhou, Z. Zhang, *Adv. Funct. Mater.* **2016**, *26* (2), 303, https://doi.org/10.1002/adfm.201503579; q) K.

J. Ji, H. H. Zhao, J. Zhang, J. Chen, Z. D. Dai, *Appl. Surf. Sci.* **2014**, *311*, 351, https://doi.org/10.1016/j.apsusc.2014.05.067; r) F. Shahzad, M. Alhabeb, C. B. Hatter, B. Anasori, S. M. Hong, C. M. Koo, Y. Gogotsi, *Science* **2016**, *353* (6304), 1137, https://doi.org/10.1126/science.aag2421; s) Y. J. Wan, P. L. Zhu, S. H. Yu, R. Sun, C. P. Wong, W. H. Liao, *Carbon* **2017**, *115*, 629, https://doi.org/10.1016/j.carbon.2017.01.054; t) Y. Yuan, W. L. Yin, M. L. Yang, F. Xu, X. Zhao, J. J. Li, Q. Y. Peng, X. D. He, S. Y. Du, Y. B. Li, *Carbon* **2018**, *130*, 59, https://doi.org/10.1016/j.carbon.2017.12.122; u) C. X. Lei, Y. Z. Zhang, D. Y. Liu, K. Wu, Q. Fu, *ACS Appl. Mater. Interfaces* **2020**, *12* (23), 26485, https://doi.org/10.1021/acsami.0c07387; v) L. Li, Y. X. Cao, X. Y. Liu, J. F. Wang, Y. Y. Yang, W. J. Wang, *ACS Appl. Mater. Interfaces* **2020**, *12* (24), 27350, https://doi.org/10.1021/acsami.0c05692; w) B. Zhou, Z. Zhang, Y. L. Li, G. J. Han, Y. Z. Feng, B. Wang, D. B. Zhang, J. M. Ma, C. T. Liu, *ACS Appl. Mater. Interfaces* **2020**, *12* (4), 4895, https://doi.org/10.1021/acsami.9b19768; x) W. T. Cao, F. F. Chen, Y. J. Zhu, Y. G. Zhang, Y. Y. Jiang, M. G. Ma, F. Chen, *Acs Nano* **2018**, *12* (5), 4583, https://doi.org/10.1021/acsnano.8b00997; y) H. W. Wei, M. Q. Wang, W. H. Zheng, Z. X. Jiang, Y. D. Huang, *Ceram. Int.* **2020**, *46* (5), 6199, https://doi.org/10.1016/j.ceramint.2019.11.087; z) Z. X. Liu, W. Y. Wang, J. J. Tan, J. Liu, M. F. Zhu, B. L. Zhu, Q. Y. Zhang, *J. Mater. Chem. C* **2020**, *8* (21), 7170, https://doi.org/10.1039/d0tc01249a; aa) Z. Y. Zhan, Q. C. Song, Z. H. Zhou, C. H. Lu, *J. Mater. Chem. C* **2019**, *7* (32), 9820, https://doi.org/10.1039/c9tc03309b; ab) D. Hu, X. Huang, S. Li, P. Jiang, *Compos. Sci. Technol.* **2020**, *188*, 107995, https://doi.org/10.1016/j.compscitech.2020.107995.
